# Linking dietary-derived arsenic exposure to blood serum arsenic burden, potential food sources, and breast cancer risk in the EPIC-Heidelberg cohort

**DOI:** 10.1186/s12885-026-17282-7

**Published:** 2026-11-12

**Authors:** Maureen Kipkoech, Celine M Beck, Anna Jaeger, Katrin Blume, Jan Lubiński, Wojciech Marciniak, Róża Derkacz, Theron Johnson, Rudolf Kaaks, Verena A Katzke

**Affiliations:** 1 Division of Cancer Epidemiology, German Cancer Research Center (DKFZ), Im Neuenheimer Feld, 69120 Heidelberg, Baden Württemberg, Germany; 2 Medical Faculty Heidelberg, Heidelberg University, Im NeuenheimerFeld, 69120 Heidelberg, Baden-Württemberg, Germany; 3 Institute for Medical Information Processing, Biometry, and Epidemiology, Chair of Public Health and Health Services Research, Faculty of Medicine, LMU Munich, Munich, Germany; 4 Pettenkofer School of Public Health, Munich, Germany; 5 Institute of Clinical Epidemiology and Biometry, Julius Maximilian University of Würzburg, Würzburg, Germany; 6 Department Exposure, German Federal Institute for Risk Assessment (BfR), Max-Dohrn-Straße 8–10, 10589 Berlin, State, Germany; 7 Department of Genetics and Pathology, Pomeranian Medical University, Powstánć ow Wlkp. 72, Szczecin, 70-204 West Pomeranian, Voivodeship Poland; 8 Read-Gene SA, ul. Alabastrowa 8, Grzepnica, 72-003 West Pomeranian, Voivodeship Poland

**Keywords:** Serum arsenic, Inorganic arsenic intake, Total arsenic intake, Breast cancer, EPIC-Heidelberg

## Abstract

**Purpose:**

Breast cancer is the most common cancer among women worldwide, yet the role of environmental toxicants remains unclear. Arsenic is an International Agency for Research on Cancer classified carcinogen linked to skin, lung, and bladder cancers. The relationship between dietary arsenic intake levels and blood arsenic concentrations, particularly across sexes, and the association between dietary arsenic intake and breast cancer remain understudied. This study aimed to address these gaps.

**Methods:**

Measurements of arsenic in foods collected from 1995 to 2022 in the German monitoring programme were used to estimate long-term dietary intake. Serum arsenic levels were measured using inductively coupled plasma mass spectrometry. Participants were aged 35–65 years; 2,674 non-cases were included in the arsenic intake-serum arsenic analysis, and 631 incident cases and 1,708 non-cases in the breast cancer analysis. Partial spearman’s rank correlation and logistic regression models assessed the association between dietary arsenic intake and serum arsenic levels. Prentice-weighted Cox proportional hazards models with age as underlying time scale were used to assess associations between dietary arsenic intake and breast cancer risk.

**Results:**

No association was observed between total or estimated inorganic arsenic intake and breast cancer risk (HR 1.1, 95% CI: 0.93–1.31; HR 0.9, 95% CI: 0.76–1.07), including in subgroup analyses by menopausal status (pre- and post-menopausal), estrogen receptor (ER), progesterone receptor (PR), and human epidermal growth factor receptor 2 (HER2) status. Weak positive correlations were observed between total and estimated inorganic arsenic intake and serum arsenic concentrations (ρ = 0.20 and ρ = 0.07). However, regression analyses showed that total arsenic intake was associated with serum arsenic in both sexes (men: OR 3.48, 95% CI 2.33–5.22; women: OR 2.60, 95% CI 1.84–3.68), whereas estimated inorganic arsenic intake were associated only among women in the second and fourth quintiles (OR 1.53 [95% CI 1.10–2.14] and 1.90 [1.33–2.73], respectively).

**Conclusion:**

Dietary arsenic intake was not significantly associated with serum arsenic levels or incident breast cancer.

**Supplementary Information:**

The online version contains supplementary material available at https://doi.org/10.1186/s12885-026-17282-7.

## Introduction

Breast cancer is the most prevalent cancer among women and a leading cause of cancer-related mortality. According to the Global Cancer Observatory (GLOBOCAN) statistics, more than two million new cases of breast cancer are diagnosed annually, underscoring the need to better understand modifiable risk factors, including dietary exposures [1]. Arsenic is a widespread environmental toxicant and a significant public health concern due to its well-established links with a range of health conditions, including skin and cardiovascular diseases, cancers, neurological disorders, and diabetes mellitus [2]. The Internal Agency for Research on Cancer (IARC) has established that arsenic exposure is associated with skin, lung and bladder cancers [3]; however, evidence linking dietary arsenic exposure specifically to breast cancer remains limited and inconsistent. Experimental studies suggest that arsenic may disrupt endocrine signaling, induce oxidative stress, and alter epigenetic regulation, thereby contributing to breast cancer [4].

An estimated 94 to 220 million people worldwide are exposed to high levels of arsenic in groundwater, far exceeding the recommended safety limit of 10 µg/L, making water the primary pathway of exposure [5–7]. Additional exposure occurs through dietary intake, industrial activities, as well as through tobacco smoke, highlighting the importance of identifying exposure sources and quantifying dietary intake [8]. To accurately assess the risk associated with each exposure route and design effective public health interventions, it is critical to understand the relative contribution of each route.

Most research on cancer and arsenic have focused on drinking water, particularly in countries identified as having high levels of arsenic in underground water, such as Argentina, Bangladesh, Cambodia, Chile, China, India, Mexico, Pakistan, the United States of America and Vietnam [8–10]. Nevertheless, in populations where water arsenic concentrations are low or regulated, food has emerged as an increasingly important source of chronic low-level arsenic exposure. Consumption of foods potentially contaminated with arsenic, such as rice-based products, seafood, non-alcoholic beverages, cereals, meat, poultry, dairy products, and certain vegetables, can substantially contribute to total dietary arsenic intake, and even chronic consumption at low levels can markedly increase biological exposure [8, 11].

Several markers, including urine, blood, hair, nails and diet via Food Frequency Questionnaires (FFQs), can be used to assess human arsenic exposure [12]. A recent meta-analysis of arsenic exposure and breast cancer reported higher arsenic levels among cases, but heterogeneity and lack of confounder adjustment limited the reliability of the findings [13]. We previously investigated serum arsenic exposure and breast cancer risk in the EPIC Heidelberg cohort. To date, only two prospective studies have examined this association: EPIC and a Polish cohort. Marciniak et al. (Polish cohort) reported a positive association [14], whereas we found no evidence of an association [15]. At the time of our previous study [15], FFQ-derived arsenic intake data were unavailable, limiting its assessment. The relationship between dietary-derived arsenic intake and blood arsenic levels remains unknown in both men and women, limiting understanding of how exposure may influence health outcomes across sexes. These gaps underscore the need for studies using both external and internal exposures in large, well-characterized populations to better assess external and internal arsenic burden and its correlations.

Therefore, this study sought to address these gaps by examining both correlations and associations between dietary arsenic intake and serum arsenic concentrations, stratified by sex, within the European Prospective Investigation into Cancer and Nutrition (EPIC)-Heidelberg cohort. It also investigated the association between dietary arsenic intake and breast cancer risk in the same cohort.

## Materials and methods

### Study characteristics

A case-cohort design nested within EPIC-Heidelberg cohort was used. The European Prospective Investigation into Cancer and Nutrition is a prospective, multicentric cohort study with up to 20 years of follow-up, designed to investigate the association between dietary intake, lifestyle factors and the risk of cancer and other chronic diseases. The EPIC-Heidelberg cohort is a sub-cohort of the larger EPIC study and includes approximately 25,546 individuals (53% women) recruited from population registries in Heidelberg and surrounding municipalities between August 1994 and October 1998. Eligible participants were women aged 35–65 years and men aged 40–65 years residing in the study area at recruitment. At baseline, data on dietary intake, health, lifestyle, socio-economic and anthropometric measurements were collected using self-administered questionnaires, interviews, and physical examinations.

The case-cohort was constructed in two steps as the number of incident breast cancer cases accumulated over follow-up time. To minimize use of biological specimens, a sub-cohort was selected from the full cohort after baseline recruitment using a two-stage, age-stratified case-cohort sampling design. The first sampling occurred in 2009, when 10% of cohort members were randomly selected. The second sampling took place in 2014, targeting 10% of members over the age of 50 who had not been included in the first sampling. The participants from both stages were combined to form the sub-cohort group. Consequently, the sub-cohort included 3,794 members. A total of 685 incidental breast cancer cases were tracked and recorded, including cases from both the sub-cohort and the larger cohort. After exclusions for insufficient biological samples, age younger than 50 years, previous malignancies other than non-melanoma skin cancer, and values in the highest or lowest 1% of energy intake and requirement, 631 cases were included in the analysis. Older participants were oversampled to enhance statistical power and reflect the higher incidence of breast cancer in this age group. Further details regarding the sampling process are available in previously published papers [15, 16]. At the baseline during recruitment in the 90s, blood samples were collected and stored at + 4°C to + 10°C for up to 24 h prior to processing after obtaining written informed consent. A detailed description of blood collection, storage procedures, and laboratory analyses for serum arsenic levels is available in previously published materials [15]. Arsenic was measured in serum samples collected at baseline, with the measurements conducted in 2024.

**Fig. 1:**
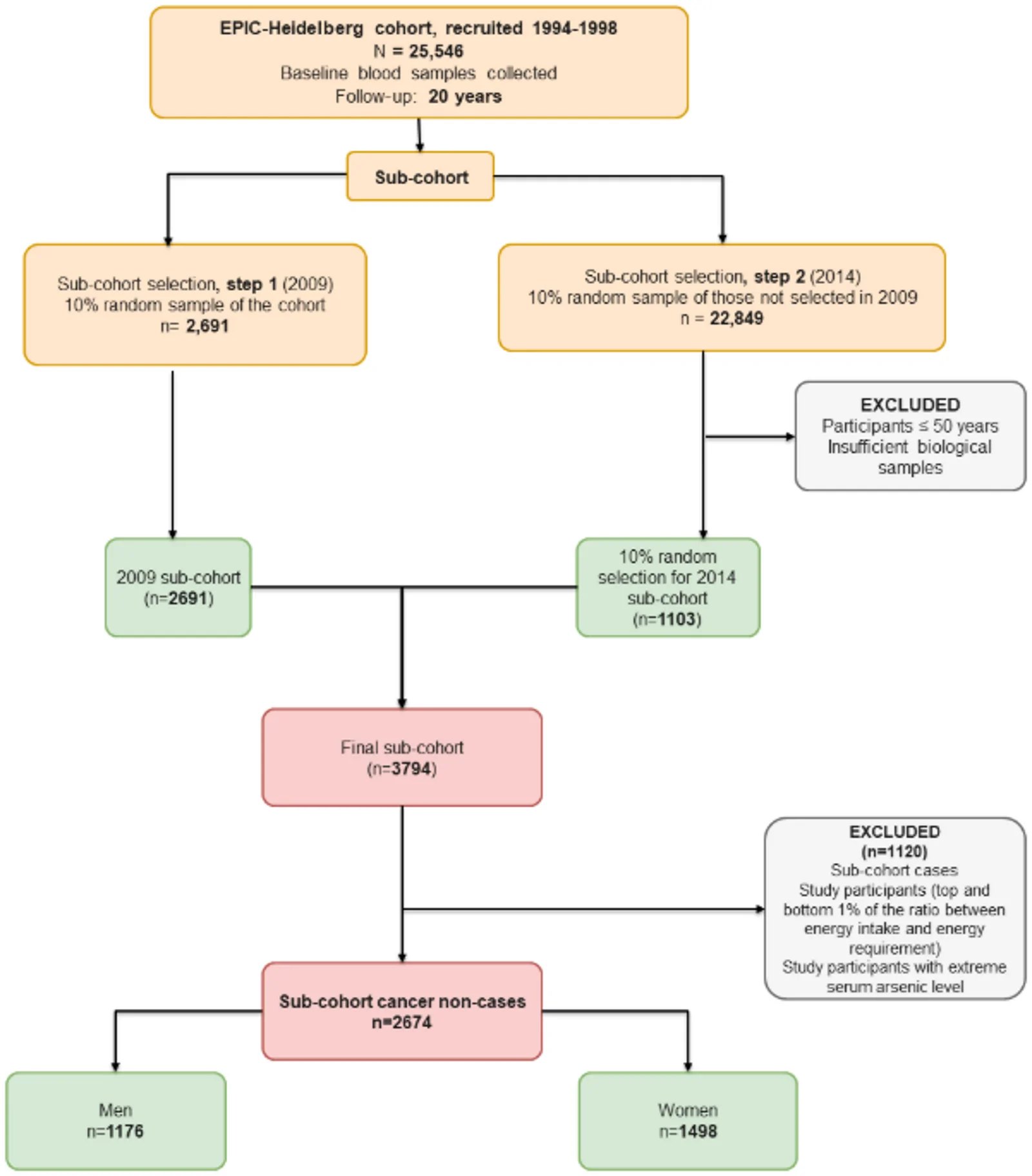
Flow chart representation of the selection process of the of EPIC-Heidelberg sub-cohort non-cases

The analysis was conducted in two parts. The first included 2,674 non-cancer sub-cohort members, excluding 50 participants with energy intake outside the recommended range or with extreme values, as well as those with unavailable blood samples [17], to examine the association between dietary arsenic intake and serum arsenic levels (Fig. 1).

The second part focused on breast cancer endpoint, comprising 631 cases and 1,708 sub-cohort non-cases. Participants with previous other malignant tumors, except non-melanoma skin cancer, were censored at the date of their previous diagnosis. Incident breast cancer cases were identified through active follow-up and verified using clinical records and linkage to cancer and pathology registries (ICD-10: C50), diagnosed up to December 2014.

### Dietary arsenic intake assessment

Dietary arsenic intake and related characteristics, including fat used in food preparation, were assessed using a self-administered FFQ comprising 148 items. The FFQ captured information on food items (portion size and consumption frequencies) and associated nutrient intake over the previous 12 months. Consumption frequency ranged from never to several times per day for predefined food items, enabling comprehensive assessment of dietary intake patterns and nutrient intake. To provide a calibration reference and evaluate relative validity in EPIC, a standard computer assisted 24 h recall interview was conducted for a representative sample of approximately 1,200 to 5,000 participants per EPIC cohort. Food and beverage intake, was recorded according to predefined meal occasions such as breakfast, lunch, dinner and snacks. Foods were quantified using a picture book specifically developed for EPIC, which displayed food in dishes of different portion sizes. This approach facilitated the collection of standardized dietary data across multiple European countries. Using standardized food recordings, the computer automatically coded participants’ food and dishes intake and calculated the corresponding nutrient intake [18]. The food frequency questionnaire portion sizes were derived from predefined standard portion assumptions [19]. In contrast, the 24 h dietary recalls independently captured actual intake using standardized dietary assessment tools. The assessed food items were categorized into food groups, potato and tubers, vegetables, legumes, fruits, dairy products, cereal and cereal products, meat and meat products, fish and shellfish, egg and egg products, fats, sugars and sweets, cakes, non-alcoholic beverages, alcoholic beverages, condiments and sauces, soups and bouillon, and miscellaneous. These groups were further subdivided into several subgroups, as shown in Supplementary Table S1, in accordance with the classification system used in the 24 h recall. Mineral water was used as a proxy matrix for the estimation of coffee, tea, and soups.

The exposure analysis was based on tAs intake data obtained during the 1995–2022 monitoring period. Between 1998 and 2022, results from 792 monitored foods could be linked to 545 food-items recorded in the 24 h dietary recalls of the EPIC study. Estimated iAs intake concentrations were based on ​EFSA calculations (2009) [20]. By these means, 100% of organic arsenic in drinking water equals iAs, 2% and 70% of tAs in fish and in all other food items respectively. The resulting estimates were used for exposure and risk assessment. Because data coverage for the 1990s was limited, BfR extended the assessment period to 2022. Monitoring data from the 1990s to 2022 showed no major discrepancies, supporting the use of data from the entire period. Therefore, all available monitoring data were included to maximize coverage and improve the matching of food-items for exposure assessment.

Data on tAs concentrations in food obtained from the National Monitoring programme in Germany was used to calculate long-term dietary exposure. In the National Monitoring, a set selection of representative food items (the so called “food basket”) is sampled in a five-year cycle. Mostly, samples are taken on the level of raw agricultural commodities (RAC) or “as purchased”, i.e., the sample consists of dry rice or raw meat. Further details on the methods used in the National Monitoring programme have been published previously [21, 22].

For the present study a set of 139,280 analytical results of arsenic from the years 1995 to 2022 was extracted from the database of the Food Monitoring Program at BfR. These were allocated to 545 food items derived from the EPIC 24 h recall questionnaire with a minimum of 20 samples per food item. Extraction of arsenic and allocation to food items was performed by statisticians of BfR. Thereafter, these 545 food items were assigned to the 148 FFQ items and weighted according to their consumption in the 24 h recall, resulting in arsenic intake per FFQ item and, subsequently, food group. This method has been previously and standardized applied to all other nutrients in the EPIC cohort [23]. The lower bound (LB) and upper bound (UB) approach was calculated for each sample. For LB, values below the quantification limit were set to the respective detection limit, whereas values below the detection limit were set to zero. For UB, values below both the quantification and detection limits were set to the respective limit.

Arsenic exposure was expressed in micrograms per kilogram of body weight per day (µg/kg bw/d) for each participant. Moreover, the arsenic exposure from the predefined food groups and their respective sub-groups was calculated using mean, median and 95th percentile concentration values in UB and LB approach, respectively, for each participant. Exposure was calculated by multiplying mean consumption amounts by corresponding mean arsenic concentrations for each food item, with results summed to derive total dietary exposure. Contributions of individual food groups to total exposure were subsequently quantified. Dietary intake from 24 h recall was assigned to the corresponding FFQ items, and arsenic exposure was estimated by linking FFQ items to concentration data and weighting by intake amounts derived from these recalls. These estimates were combined with mean arsenic concentrations measured at the food item level. For reporting purposes, results were aggregated into broader food groups including fish, meat, vegetables, cereals, fruit, and beverages due to large number of food groups (*n* = 545). A limited number of subgroups were analyzed separately, including within cereals, where rice and bread/bakery products were distinguished. Participants in the top and bottom 1% of the ratio between energy intake and energy requirement were excluded.

### Statistical analysis

The baseline characteristics and subsequent analyses were based on the median upper bound values of both total and estimated inorganic arsenic intake. The upper bound was selected to represent a conservative scenario for arsenic concentrations, in line with literature [24]. The choice of summary statistic represents distinct methodological decision. A total of 2,674 non-cases from the sub-cohort were included in this study. Continuous variables were summarized as median and interquartile range (IQR), while categorical variables were summarized using frequencies and percentages. Histograms and density curves were generated to assess the distribution of dietary arsenic intake and serum arsenic levels. The food sources contributing to tAs intake and estimated iAs intake were presented using bar charts. Partial spearman’s rank correlation coefficients were used to assess correlation between dietary arsenic intake and serum arsenic levels across food groups and their subgroups. Serum arsenic was modelled as a continuous outcome to assess its correlation with dietary arsenic intake. Analyses were stratified by sex and adjusted for age at recruitment. The 95% confidence intervals for the partial spearman correlation coefficients were calculated from 5,000 non-parametric bootstrap resamples to obtain stable estimates. Generalized linear model was used to assess the relationship between dietary arsenic intake levels and breast cancer-related factors. Arsenic levels were analyzed on their original scale using a gamma regression model with a log link. Multiplicative effects were obtained by exponentiating regression coefficients (β), with the resulting estimates expressed as percentage changes using the formula [exp(β) − 1] × 100. Both dietary arsenic intake and serum arsenic concentrations were assessed as outcomes. Weighting was not applied because the analysis was restricted to the randomly selected sub-cohort, comprising 1,708 non-cases and 99 breast cancer cases among women. Dietary arsenic intake was categorized into quintiles (food groups) to assess exposure gradients, with the lowest exposure category serving as the reference group. Serum arsenic concentrations, the outcome, was dichotomized at the median to assess whether the odds of above-median concentrations increased across dietary intake quintiles. Odds ratios were estimated using logistic regression models with 95% confidence intervals adjusted for covariates and stratified by sex. The statistical power to detect an association between serum arsenic concentrations and incident breast cancer was estimated to exceed 80% for a hazard ratio of 1.4 when comparing women with arsenic concentrations above versus below the median, assuming a significance level of α = 0.05. A prentice weighted Cox proportional hazard regression model, using age as the underlying time scale, was employed to estimate hazard ratios (HRs) and 95% confidence intervals for breast cancer, based on dietary arsenic intake levels. All members of the randomly selected sub-cohort were assigned a weight of 1, irrespective of breast cancer status, as were participants with breast cancer who were outside the sub-cohort. All other participants were assigned a weight of 0 and did not contribute to the analysis. Crude and adjusted prentice weighted Cox proportional hazard regression model were constructed. A knowledge-based directed acyclic graph (DAG) was used to depict the presumed relationships between dietary arsenic intake and breast cancer to guide a priori covariate selection. Common causes of dietary arsenic intake and breast cancer were considered potential confounders and included in multivariable models. Potential mediators were excluded from the primary adjustment set, whereas variables included mainly to improve precision were distinguished from confounders. All sub-cohort observations were subject to left truncation at recruitment age. During follow-up, observations were right-censored based on whichever event occurred first: incident breast cancer, loss to follow-up or death. Model adjustment accounted for level of education (primary, secondary, technical/professional, tertiary), smoking status (never, former, current, unknown), alcohol use (never, former, current at recruitment, lifetime), body-mass index (BMI, continuous), fruit and vegetable intake (continuous), physical activity (inactive, moderately inactive, moderately active, active) parity status (nulliparous, primiparous, multiparous), use of oral contraceptives or hormone replacement therapy (HRT), breastfeeding history (yes, no), age at menarche (< 12 years, 12–14, > 14 years), and family history of breast cancer (yes, no). Analyses of individual food sources in relation to serum arsenic concentrations, and subgroup analyses of dietary arsenic intake according to estrogen receptor (ER), progesterone receptor (PR)​, human epidermal growth factor receptor 2 (HER2) status, and menopausal status at baseline and diagnosis, were considered exploratory. Sensitivity analyses were conducted using the median lower bound and the mean lower and upper bounds of dietary arsenic intake to assess the robustness of the findings. Analysis was performed using R software (version 4.5.1, R Project for Statistical Computing, RRID: SCR_001905, Posit Software, PBC, Boston, MA, USA available under the GNU Affero General Public License version 3 (AGPL v3)) and SAS version 8.3 (version 8.3; SASCorp LLC, 2019–2020, Cary, NC, USA).

## Results

### Participants characteristics

Dietary arsenic intake and serum arsenic levels were non-normally distributed (Supplementary Figures S1, S3, and S5); however, after log₂ transformation, the values approximated a normal distribution. The median tAs intake was 0.59 µg/kg bw/d, while the estimated median iAs intake was considerably lower at 0.23 µg/kg bw/d (Table 1). The blood serum arsenic levels were 0.83 µg/L. Across all measurements, sex-related differences in both dietary arsenic intake and serum arsenic concentrations were minimal. The median age at recruitment was 52.7 years, and the median BMI was 25.5 kg/m², with men tending to have a slightly higher BMI compared to women. At least three quarter had attained a secondary school level of education or higher. A larger proportion (40%) were classified as moderately active in physical activity, a pattern observed in both males (33%) and females (46%). Most participants reported never having smoked (47%), with notable differences between sexes: the majority of males (42%) were former smokers, whereas most females had never smoked (55%). At recruitment, alcohol consumption was higher among men, with a median of 19.37 g/d compared to 5.20 g/d among women.

**Table 1:** Baseline characteristics of the sub-cohort non-cases stratified by sex in EPIC-Heidelberg cohort (*N* = 2,674)

Variable	Sub-cohort non cases	Men	Women
Arsenic (median, IQR)	(*n* = 2,674)	44% (*n* = 1,176)	56% (*n* = 1,498)
Total arsenic intake UB (µg/kg bw/d)	0.59 (0.42–0.80)	0.59 (0.42–0.80)	0.59 (0.42–0.79)
Estimated iAs intake UB (µg/kg bw/d)	0.23 (0.18–0.28)	0.22 (0.18–0.27)	0.23 (0.18–0.29)
Blood serum arsenic (µg/L)	0.83 (0.59–1.28)	0.85 (0.61–1.31)	0.82 (0.58–1.24)
Age at recruitment (median, IQR)			
Age at recruitment (years)	52.70 (45.61–57.89)	53.83 (47.36–58.21)	51.83 (43.04 57.50)
General anthropometry (median, IQR)			
Body Mass Index (kg/m2)	25.48 (22.93–28.17)	26.33 (24.27–28.53)	24.44 (22.02–27.79)
Highest school level attained			
Primary education or less	724 (27%)	316 (27%)	408 (27%)
Secondary	184 (7%)	63 (5%)	121 (8%)
Technical	931 (35%)	310 (26%)	621 (42%)
Tertiary	835 (31%)	487 (42%)	348 (23%)
Physical activity			
Inactive	554 (21%)	303 (26%)	251 (17%)
Moderately inactive	795 (30%)	358 (30%)	437 (29%)
Moderately active	1078 (40%)	390 (33%)	688 (46%)
Active	247 (9%)	125 (11%)	122 (8%)
Smoking status			
Never	1251 (47%)	435 (37%)	816 (55%)
Former	899 (34%)	493 (42%)	406 (27%)
Smoker	517 (19%)	244 (21%)	273 (18%)
Unknown	7 (0)	4 (0)	3 (0)
Alcohol consumption (median, IQR)			
Alcohol at recruitment (g/d)	10.03 (2.84–24.13)	19.37 (7.98–37.68)	5.20 (1.75–16.03)

### Arsenic concentrations in foods from 1995 to 2022 (median [IQR]) µg/100 g LM

More than 500 food items were compiled to estimate dietary arsenic intake across 17 food groups. Composite values for arsenic were derived for major food groups and their principal sub-groups (453). For each food group, item-level median upper-bound arsenic concentrations were pooled across all food items, and the group median and IQR were calculated from the resulting distribution (Table 2). Fish and shellfish had the highest overall median levels (123 [79.5]), driven by fish (116 [82.5]) and fish products and fish in the crumbs (140 [16.5]). In contrast, cereal and cereal products showed much lower median concentrations (1.96 [1]), with rice (2[2.1]) and pasta and other grains (2 [0]) contributing similar values. Sugar and confectionary had a comparable median level of 2 (1). Dairy products showed modest concentrations (1.25 [1]), with milk (1.1 [0.1]) and yoghurt (3 [2]). Non-alcoholic beverages were low overall (0.6 [0.6]), including roasted coffee infusion (1 [0]), while mineral water had the lowest level (0.1 [0]). Alcoholic beverages also demonstrated low median arsenic intake levels (1 [1]), with beer and cider negligible 0.81 (0.38) and wine at 2 (1).

**Table 2:** Dietary arsenic intake concentrations assigned to food groups in the 1990s (median [IQR]) µg/100 g LM

Food group	Food items	Sub-group	Arsenic concentration median upper bound (IQR)
Potatoes and other tubers	11		1(0.3)
Vegetables	46		1(0)
Legumes	16		1(0.38)
Fruits	45		0.7(0.41)
Dairy products	35		1.25(1)
		Milk	1.1(0.1)
		Yorghurt	3[25]
Cereals and cereal Products	55		1.96(1)
		Rice	2(2.1)
		Pasta and other grains	2(0)
Meat and meat products	88		1(0.01)
		Processed Meat	1(0.04)
Fish and shellfish	20		123(79.5)
		Fish	116(82.5)
		Fish products, fish in crumbs	140(16.5)
Eggs and egg products			0.42(0)
Fat	24		2(0.8)
Sugar and confectionary	18		2(1)
Cakes and biscuits	24		1(0)
Non-alcoholic beverages	16		0.6(0.6)
		Roasted coffee infusion	1(0)
		Mineral water	0.1(0)
Alcoholic beverages	11		1(1)
		Wine	2(1)
		Beer, cider	0.81(0.38)
Condiments and sauces	27		1(0.39)
Soups, bouillons	13		0.52(0.33)
Miscellaneous	4		1.5(2.10)

### Dietary sources of total and estimated inorganic arsenic intake across food groups (µg/kg bw/d), stratified by sex

**Fig. 2:**
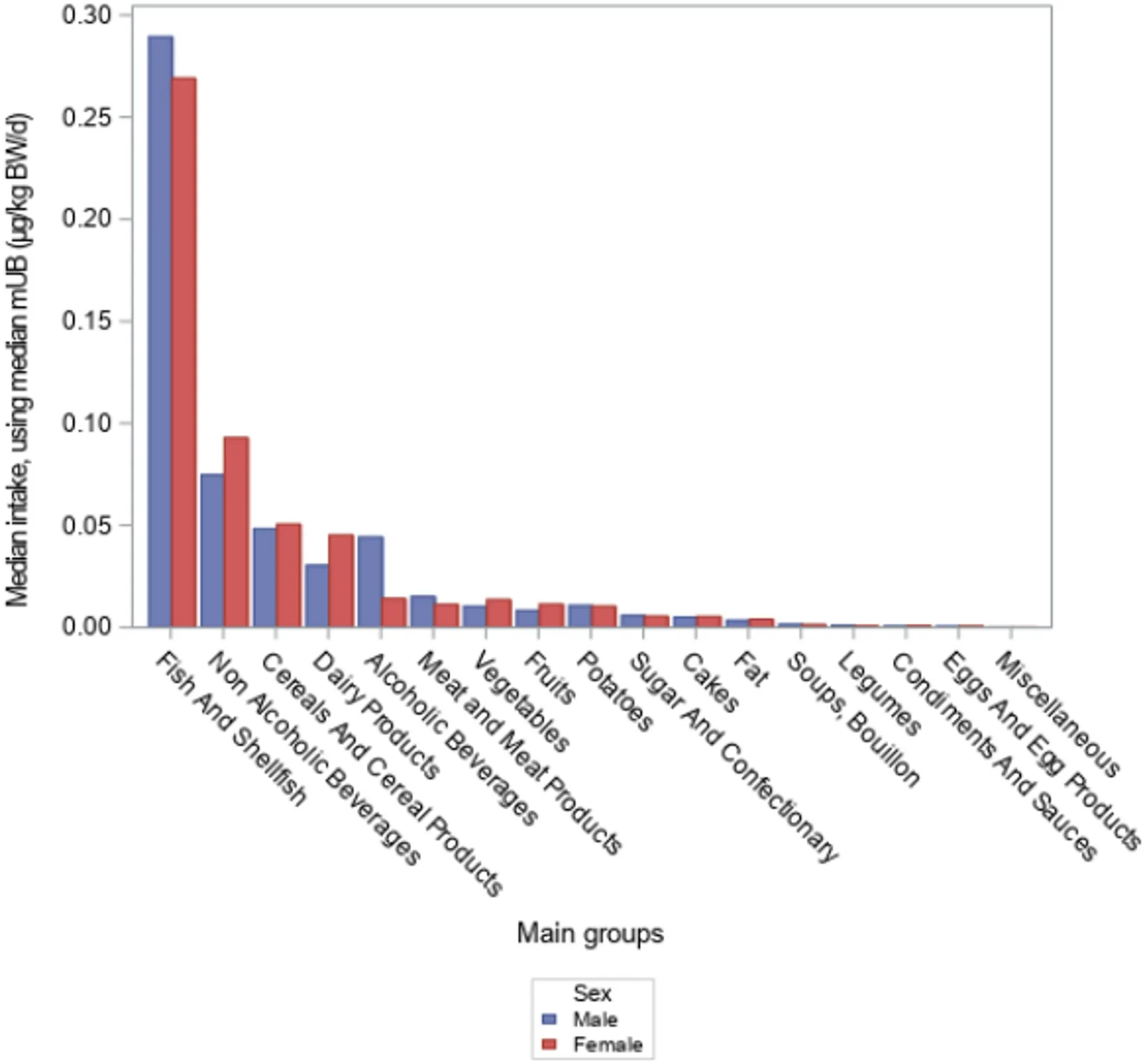
Median tAs intake across 17 food groups, stratified by sex

Figure 2 presents the distribution of food groups contributing to tAs intake among men and women. The highest tAs intake was observed in fish and shellfish for both men (0.29 [0.36]) and women (0.27 [0.30]). This was followed by non-alcoholic beverages and cereal and cereal products. Among non-alcoholic beverages, median levels were 0.07 (0.1) in men and 0.09 (0.13) in women, with coffee being the largest contributor (men: 0.04 (0.05) ; women: 0.05 (0.06)). Within the cereal and cereal products, median levels were 0.05 (0.07) in both men and women, with rice contributing the highest levels 0.02 (0.02). For estimated iAs intake (Supplementary Figure S7), non-alcoholic beverages contributed the highest intake for both men (0.05 [0.08]) and women (0.07 [0.09]), with coffee being the main contributor in both sexes (0.03 [0.04]). This was followed by cereal and cereal products, contributing 0.03 (0.05) among men and 0.04 (0.05) among women, with rice being the principal contributor in both sexes (0.01 [0.01]). The third highest contributing food group differed by sex: among men, alcoholic beverages contributed 0.03 (0.04), led by beer and cider 0.01 (0.02), whereas among women, dairy products contributed 0.031 (0.05), with milk and yogurt accounting 0.01 (0.01) and 0.01 (0.02), respectively.

### Association between dietary arsenic intake (µg/kg bw/d) and serum arsenic concentrations (µg/L), stratified by sex

Supplementary Table S4 presents the partial spearman’s rank correlation coefficients and corresponding p-values for tAs intake and estimated iAs intake in relation to serum blood arsenic concentrations, adjusted for sex and age at recruitment. Total arsenic intake was weakly but significantly correlated with serum arsenic: ρ = 0.20 (95% CI: 0.17–0.24; *p* < 0.0001) for both sexes combined, ρ = 0.20 (95% CI: 0.14–0.25; *p* < 0.0001) for men, and ρ = 0.21 (95% CI: 0.16–0.26; *p* < 0.0001) for women. Estimated iAs intake showed similarly positive weak correlations: ρ = 0.07 (95% CI: 0.03–0.11; *p* < 0.0001 for both sexes combined, ρ = 0.07 (95% CI: 0.01–0.13; *p* = 0.02) for men, and ρ = 0.07 (95% CI: 0.02–0.12; *p* = 0.01) for women. Correlations between food groups with serum arsenic levels are provided in the Supplementary Table S5. Logistic regression results (Table 3) showed that tAs intake was associated with increased odds of serum arsenic above the median in both men and women. Men in the fifth intake group had significantly higher odds compared with the first group (OR 3.48, 95% CI 2.33–5.22), and women in the highest group also had elevated odds (OR 2.6, 95% CI 1.84–3.68). For estimated iAs intake, the pattern differed. Among men, only intake group 5 was significantly associated with blood serum arsenic in the crude model (OR 1.57, 95% CI 1.09–2.27), but this lost significance after adjustment. Among women, significant associations were observed only in the second and fourth quintiles, with the highest odds in the fourth quintile (OR 1.90, 95% CI 1.33–2.73). There was no evidence of sex-specific differences in the associations of tAs intake and estimated iAs intake with serum arsenic concentrations (P for interaction = 0.46 and 0.05, respectively).

**Table 3:** Association between dietary arsenic intake and serum arsenic concentrations in the EPIC-Heidelberg case–cohort (OR and 95%CI)

	Food groups divided into quintiles					*p*-trend
	Q1	Q2	Q3	Q4	Q5	
Total arsenic intake: Men						
Sub-cohort non-cases	235	235	235	235	236	
Crude OR (95% CI)	Ref	1.70 (1.17–2.47)	2.01 (1.39– 2.93)	1.79 (1.24 − 2.60)	3.71(2.54–5.45)	< 0.001*
Adjusted OR (95% CI)^a^	Ref	1.63 (1.12–2.38)	1.94 (1.33–2.85)	1.73 (1.18–2.54)	3.48(2.33–5.22)	< 0.001*
Total arsenic intake: Women						
Sub-cohort non-cases	300	299	300	299	300	
Crude OR (95% CI)	Ref	1.33(0.96– 1.84)	1.63 (1.18–2.26)	1.98 (1.43– 2.75)	2.70(1.95–3.76)	< 0.001*
Adjusted OR (95% CI)^a^	Ref	1.31(0.94–1.82)	1.61 (1.16–2.24)	1.94(1.39–2.72)	2.60(1.84–3.68)	< 0.001*
Estimated iAs intake: Men						
Sub-cohort non-cases	235	235	235	235	236	
Crude OR (95% CI)	Ref	1.09 (0.76–1.57)	0.95(0.66–1.37)	1.27(0.88–1.83)	1.57(1.09–2.27)	0.01*
Adjusted OR (95% CI)^a^	Ref	1.00 (0.69–1.45)	0.80(0.54–1.17)	1.01(0.67–1.51)	1.23(0.80–1.88)	0.4
Estimated iAs intake: Women						
Sub-cohort non-cases	300	299	300	299	300	
Crude OR (95% CI)	Ref	1.53(1.11–2.11)	1.46(1.06–2.02)	2.03(1.47–2.82)	1.46(1.06–2.02)	< 0.001*
Adjusted OR (95% CI)^a^	Ref	1.53(1.10–2.14)	1.41(1.00–1.98)	1.90 (1.33–2.73)	1.34(0.92–1.96)	0.07

^a^Adjusted for age at recruitment, level of education, BMI, smoking status, and alcohol use

### Associations between breast cancer-related factors and arsenic levels among women

Using a generalized linear model (Table 4), we examined how breast cancer-related factors were associated with arsenic levels. Each one-unit increase in BMI was inversely associated with 3.15% and 1.95% lower estimated iAs and tAs intake levels, respectively (iAs: -3.15, 95% CI -3.45 to -2.85; tAs: -1.95, 95% Cl -2.62 to -1.26), and was also inversely associated with serum arsenic, although this was not statistically significant (-0.55, 95% Cl -1.75 to 0.68). Secondary education was associated with tAs intake, with a 25.57% higher level compared with primary education (25.57, 95% CI 10.20 to 43.52). Physically active women had 9.82% higher tAs intake levels (tAs: 9.82, 95% Cl -1.20 to 21.97) and 2.34% higher serum arsenic levels (As: 2.34, 95% Cl -14.08 to 21.65); however neither was statistically significant. In contrast, physically active women had 5.48% higher estimated iAs intake levels (iAs: 5.48 95% Cl 0.63 to 10.53), which was statistically significant. Current smokers had 8.31% higher tAs intake levels compared with never smokers (-8.31,95% Cl -0.42 to 17.93%), and 12.39% higher serum arsenic levels (12.39, 95% Cl -2.23 to 29.54), while estimated iAs intake was statistically significant at 7.16%, 95% Cl 3.21 to 11.29. Each one-unit increase in alcohol consumption was associated with 0.64%, and 0.61% higher estimated iAs and tAs intake levels, respectively, and 0.62% higher serum arsenic levels (iAs: 0.64, 95% CI 0.54 to 0.74; tAs: 0.61, 95% Cl 0.38 to 0.83; serum: 0.62, 95% Cl 0.24 to 1.01). HRT use was significantly associated with 14.44% higher serum levels (14.44, 95% Cl: 2.39 to 28.02). Age at recruitment, parity status, age at menarche, breastfeeding and family history of breast cancer were unrelated to both dietary and serum arsenic levels.

**Table 4:** Associations between breast cancer-related factors and arsenic levels in the EPIC-Heidelberg sub cohort non-cases (*N* = 1,807)

	Estimated inorganic arsenic intake			Total arsenic intake			Serum arsenic		
Variable	Relative Change	Lower 95% CI	Upper 95% CI	Relative Change	Lower 95% CI	Upper 95% CI	Relative Change	Lower 95% CI	Upper 95% CI
Intercept	-48.71	-55.08	-41.42	-18.61	-39.37	9.40	-22.27	-52.78	28.35
BMI (continuous)	-3.15	-3.45	-2.85	-1.95	-2.62	-1.26	-0.55	-1.75	0.68
Age at recruitment	-0.09	-0.27	0.10	0.26	-0.15	0.68	0.61	-0.09	1.32
Alcohol intake (g/d)	0.64	0.54	0.74	0.61	0.38	0.83	0.62	0.24	1.01
Parity status (ref: Nulliparous)									
Primiparous	0.85	-4.33	6.33	-1.40	-12.43	11.09	0.73	-17.15	22.65
Multiparous	0.70	-4.37	6.05	0.86	-10.19	13.32	11.91	-7.60	35.67
Grand multiparous	1.94	-10.30	16.26	-5.46	-28.84	27.80	10.27	-30.61	83.71
Age at menarche (ref: early ≤ 12 years)									
Average (13–14)	-2.33	-5.32	0.74	-3.94	-10.41	2.97	-4.52	-14.98	7.13
Late (≥ 15)	-0.51	-4.58	3.75	6.45	-3.08	17.01	7.46	-8.09	25.92
Breastfeeding (ref: no)									
Yes	0.63	-3.34	4.73	1.10	-7.71	10.58	1.84	-12.50	18.04
Education (ref: Primary)									
Secondary	2.03	-3.77	8.26	25.57	10.20	43.52	-0.18	-19.56	24.85
Technical/Professional	-0.82	-4.12	2.58	-3.47	-10.54	4.12	4.19	-8.09	18.03
Tertiary	-0.59	-4.75	3.76	0.44	-8.75	10.59	8.43	-7.46	27.12
Oral contraceptive/HRT use (ref: no)									
Yes	-1.62	-4.49	1.34	-1.48	-7.85	5.36	14.44	2.39	28.02
Family history of breast cancer (ref: no)									
Yes	2.78	-2.66	8.62	4.16	-7.74	18.08	-11.29	-27.28	9.44
Unknown	1.29	-2.28	5.02	3.36	-4.62	12.15	-6.08	-17.75	7.60
Smoking status (ref: Never smoker)									
Former smoker	-0.30	-3.53	3.05	0.16	-6.98	7.89	4.30	-7.59	17.89
Current smoker	7.16	3.21	11.29	8.31	-0.42	17.93	12.39	-2.23	29.54
Unknown	-8.97	-28.99	19.25	2.71	-39.64	95.15	-13.91	-62.58	168.63
Physical activity (ref: Inactive)									
Moderately inactive	-1.30	-5.46	3.02	0.59	-8.71	10.70	1.35	-13.82	18.77
Moderately active	0.07	-4.33	4.64	5.78	-4.39	16.92	-7.62	-21.90	8.97
Active	5.48	**0.63**	**10.53**	9.82	-1.20	21.97	2.34	-14.08	21.65

### Association between dietary arsenic intake and breast cancer risk

Table 5 shows the relationship between dietary arsenic intake and breast cancer risk. For tAs intake, participants in the highest quartile (Q4) had a higher risk of breast cancer compared with the lowest quartile (Q1) (HR 1.1, 95% Cl: 0.93–1.31), with Q2 and Q3 also indicating increased risk (Q2: HR 1.08, 95% Cl: 0.9–1.29; Q3: HR 1.03, 95% Cl: 0.86–1.22). None of these associations were statistically significant. Similarly, no clear association was observed across increasing quartiles of estimated iAs intake, with HRs of 1.02 (Q2, 95% Cl: 0.83–1.25), 0.94 (Q3, 95% Cl: 0.79–1.12), and 0.9 (Q4, 95% Cl: 0.76–1.07), respectively. Results stratified by menopausal status at baseline showed differing patterns (Supplementary Table S6). For estimated iAs intake, premenopausal women in the highest quartile had lower hazards of breast cancer in both crude and adjusted models, although not statistically significant (Crude HR Q4: 0.77, 95% CI 0.58–1.03; Adjusted HR Q4: 0.8, 95% CI 0.6–1.07). In contrast, postmenopausal women showed higher hazards in both models (Crude HR Q4: 1.09, 95% CI 0.86–1.38; Adjusted HR Q4: 1.05, 95% CI 0.83–1.33). For tAs intake, premenopausal women had higher hazards in both models (Crude HR Q4: 1.13, 95% CI 0.84–1.51; Adjusted HR Q4: 1.1, 95% CI 0.81–1.48). A similar pattern was observed among postmenopausal women (Crude HR Q4: 1.02, 95% CI 0.8–1.3; Adjusted HR Q4: 1.02, 95% CI 0.79–1.3), although none of these associations reached statistical significance. Stratification by menopausal status at diagnosis showed similar patterns for both estimated iAs and tAs intake, with no statistically significant associations. For estimated iAs intake, premenopausal women had identical crude and adjusted hazard ratios of 0.87 (95% CI 0.63–1.21). Among postmenopausal women, hazard ratios were 1.01 (95% CI 0.84–1.21) in the crude model and 0.98 (95% CI 0.82–1.17) after adjustment. For tAs intake, premenopausal women had hazard ratios of 0.95 (95% CI 0.67–1.34) and 0.93 (95% CI 0.66–1.31), in crude and adjusted models, respectively. Among postmenopausal women, hazard ratios were similar in both models (1.1, 95% CI 0.92–1.32). Sensitivity analyses were conducted as detailed in the supplementary material, using the median lower bound and both the lower and upper bounds of mean dietary arsenic intake. Stratified analyses were further performed according to EP, PR and HER2 status in Supplementary Tables S8 and S9, respectively. These analyses yielded results consistent with the primary analysis, as the observed associations remained statistically non-significant. Competing events were accounted for, and the association between dietary arsenic exposure and all-cause mortality was assessed, as presented in Supplementary Table S12.

**Table 5:** Association between dietary arsenic intake and breast cancer in the EPIC-Heidelberg case–cohort (HR and 95%CI)

	Quartile of arsenic				*p*-trend
	Q1	Q2	Q3	Q4	
Total arsenic intake					
Number of cases	145	167	151	168	
Sub-cohort non-cases	422	432	430	424	
Median arsenic (µg/kg bw/d)	0.33	0.50	0.67	0.96	
Crude HR (95% CI)	Ref	1.07 (0.9–1.27)	1.02 (0.86–1.21)	1.1 (0.93–1.3)	0.38
Adjusted HR (95% CI)^a^	Ref	1.08 (0.9–1.29)	1.03 (0.86–1.22)	1.1 (0.93–1.31)	0.54
Estimated iAs intake					
Number of cases	140	156	177	158	
Sub-cohort non-cases	431	425	426	426	
Median arsenic (µg/kg bw/d)	0.15	0.20	0.25	0.33	
Crude HR (95% CI)	Ref	1.03 (0.87–1.23)	0.94 (0.79–1.11)	0.92 (0.78–1.09)	0.8
Adjusted HR (95% CI)^a^	Ref	1.02 (0.83–1.25)	0.94 (0.79–1.12)	0.9 (0.76–1.07)	0.77

^a^Adjusted for level of education, BMI, smoking status, alcohol use, physical activity, vegetable and fruit intake, parity status, use of oral contraceptives/HRT, breastfeeding history, age at menarche, and family history of breast cancer

## Discussion

Arsenic exposure has been hypothesized to play a role in the development of breast cancer. In our earlier work within the EPIC-Heidelberg cohort, we assessed this association using serum arsenic as the exposure measure; however, no statistically significant relationship was observed. Building on this, we sought to examine the question from a different perspective by using dietary-derived arsenic as an alternative exposure metric. Despite the availability of both tAs and estimated iAs intake, our findings were consistent with the earlier analysis, with no evidence of a statistically significant association with breast cancer. To further contextualize these results, we extended the analysis to characterize potential dietary sources of arsenic and to examine the relationship between dietary arsenic intake and serum arsenic levels, where a weak positive correlation was observed.

The main sources of tAs intake were fish and shellfish, whereas estimated iAs intake primarily originated from non-alcoholic beverages and cereal products. Among those beverages and cereals, coffee and rice were the key contributors, respectively, with only minor sex-specific differences observed in other food groups. These findings align with the initial BfR MEAL study, which identified rice and rice-based products as major contributors to iAs intake; fish and seafood as primary sources of tAs intake [25]. Updated risk assessments from the European Food Safety Authority’s (EFSA) [20] further support this pattern, confirming rice and cereal products among the leading dietary sources of iAs intake [26]. Together, these findings indicate consistent food-related arsenic exposure patterns across studies and over time in Europe, likely reflecting the frequent consumption of these foods within prevailing dietary practices. A review in Spain indicated that dietary arsenic exposure patterns differ across populations, reflecting local dietary habits and food choices, which shape the contribution of individual foods to overall exposure [27]. Minor sex-specific contributions, such as alcoholic beverages in men, are likely driven by differences in consumption patterns, as population studies consistently show that men, on average, consume alcoholic beverages more frequently and in larger quantities than women [28].

Serum arsenic concentrations differed modestly by sex, with males (0.85 µg/L) showing slightly higher levels than females (0.82 µg/L). In a community-based cohort investigating multiple elements in plasma, sex-related differences were reported across several elemental concentrations; however, arsenic values were not stratified by sex, limiting direct comparison with our findings [29]. A study conducted among Spanish adults reported a medium plasma arsenic concentration of approximately 1.49 µg/L, higher than the overall medium level observed in our study (0.83 µg/L) [30]. The observed differences between sexes in our study may reflect sex-dependent variation in arsenic metabolism. Women exhibit higher methylation efficiency, leading to increased conversion of iAs into dimethylarsenic acid (DMA) [31], which is more readily excreted [32]. Mechanistically, estrogen may indirectly enhance methyl donor availability and modulate the activity of key methylating enzymes, thereby facilitating more efficient arsenic biotransformation in women [33]. These mechanisms may help explain why serum arsenic concentrations were slightly higher among men than women in this study [26]. Our estimate should, however, be interpreted with caution. Because it was derived using an upper-bound approach, it represents a conservative estimate of dietary exposure rather than a precise measure of actual intake. Median estimated iAs and tAs intakes were 0.23 (IQR 0.18–0.28) and 0.59 (IQR 0.42–0.80) µg/kg bw/d, respectively. The 90th percentiles were 2.26 µg/kg bw/d per day for estimated iAs intake and 3.34 µg/kg bodyweight per day for tAs intake, indicating considerable variability in estimated dietary arsenic intake among participants. According to EFSA, the reported range for high-exposure adult consumers in Europe is 0.07–0.33 µg/kg body weight/day, within which the median upper-bound estimate of iAs intake in our study (0.23 µg/kg body weight/day) falls. Based on skin cancer as the critical health endpoint, a reference point for risk characterization was established at BMDL₀₅ of 0.06 µg/kg body weight/day [26]. Accordingly, the margin of exposure (MOE) for the estimated arsenic intake in this study was approximately 0.26 (0.06/0.23). This low MOE may indicate a potential health concern. However, the estimate should be interpreted cautiously, as it represents a conservative exposure scenario rather than a precise measure of actual intake, given that iAs intake was estimated based on tAs intake.

A positive but weak correlation was observed between both tAs and estimated iAs intake and serum arsenic concentrations (ρ = 0.20 and ρ = 0.07, respectively). However, regression analyses showed that tAs and estimated iAs intake was associated with serum arsenic in both men and women (men: OR 3.48, 95% CI 2.33–5.22; women: OR 2.6, 95% CI 1.84–3.68), whereas estimated iAs intake was associated among women only in the second and fourth quintiles, with the highest odds in the fourth quintile (OR 1.90, 95% CI 1.33–2.73). Nonetheless, the magnitude of the associations did not increase monotonically across quintiles. Differences in the exposure windows might partly explain the weak correlations between dietary arsenic intake and serum arsenic concentrations. Dietary arsenic intake derived from the FFQ reflect habitual dietary exposure over a longer period, whereas serum arsenic concentrations reflect more recent exposure and are influenced by absorption, metabolism, and elimination. Thus, a single serum arsenic measurement may not reflect long-term dietary arsenic intake, and the weak correlation may indicate that the two measures capture different aspects of arsenic exposure rather than poor validity of the dietary arsenic intake assessment. Bommarito et al. 2019 reported a moderate positive correlation between plasma arsenicals and iAs in drinking water in Italy (ρ = 0.41) [34], consistent with our findings. However, because their study did not investigate dietary arsenic intake specifically, differences in exposure sources may limit direct comparison with the correlations observed in the present research. Although previous studies have suggested an association between dietary arsenic intake and blood arsenic levels, the relationship between dietary arsenic intake and serum arsenic concentrations has been largely overlooked.

Many studies examining arsenic intake and biomarkers have focused on urinary arsenic, often including speciation of iAs, monomethylarsonic acid (MMA), and DMA [34, 35]. These urinary-based studies help contextualize the weak correlations observed in blood serum. In a cohort study of 150 Japanese adults, Yoshinaga and Narukawa (2020) observed a positive correlation between iAs intake and urinary MMA concentrations was observed (ρ = 0.544 and = 0.458 for creatinine-corrected and specific gravity–corrected values, respectively) [35], suggesting that urinary arsenic biomarkers, particularly across speciated measures, may capture recent dietary iAs exposure more sensitively than serum tAs.

Moreover, interindividual variability in urinary arsenic species has been linked to dietary sources, particularly in relation to seafood consumption, which predominantly increases organic arsenic species such as arsenobetaine and may subsequently influence urinary biomarker patterns [34]. Although these findings are not directly comparable with serum concentrations due to differences in kinetics and biological matrices, they provide important context for interpreting interindividual variability and the modest strength of dietary-blood biomarker correlations. Blood serum arsenic reflects the combined contribution of inorganic and organic species and functions as a dynamic biomarker of recent exposure [30, 36]. However, in the absence of speciation analysis, it is not possible to distinguish toxicologically relevant inorganic forms from largely non-toxic compounds, such as arsenobetaine, or to directly infer metabolic processing pathways [3, 37].

Unlike iAs intake, high intake of organic arsenic may substantially increase serum tAs concentrations without necessarily reflecting the internal toxicological burden [27, 37]. In plasma, iAs constitutes only a small fraction of tAs and is metabolized to MMA and DMA, whereas organic arsenic compounds are quickly excreted unchanged [3, 26, 37], which may explain the low correlation observed for estimated iAs intake (ρ = 0.07). Accordingly, weaker correlations between long-term dietary arsenic intake and serum arsenic concentrations would be expected given these kinetic differences. Furthermore, pronounced interindividual differences in arsenic metabolism, including sex-specific methylation efficiency, genetic polymorphisms in arsenic (+ III oxidation state) methyltransferase (AS3MT), and nutritional factors influencing methyl donor availability, can further weaken associations between intake and plasma concentrations [32, 38, 39]. Dietary arsenic intake was estimated using a FFQ, reflecting habitual intake for the past 12 months, whereas serum arsenic represents recent exposure [38, 40]. This temporal mismatch may explain the weak correlations observed between both tAs and estimated iAs intake and serum arsenic concentrations.

We did not observe any significant association between either tAs or estimated iAs intake and breast cancer. These findings were consistent across ​sensitivity analyses of breast cancer risk according to menopausal status​ at recruitment and at diagnosis, as well as ER, PR and HER2 statu, and were ​in line with our previous results on serum arsenic and breast cancer in the EPIC-Heidelberg cohort [15]. Conversely, serum arsenic reflects current exposure, limiting the ability to capture long-term accumulation; however, the concordance of findings across these two-exposure metrics strengthens the internal validity of our results. We found no evidence of an association between the dietary arsenic intake measures and breast-cancer incidence in this cohort; modest associations cannot be excluded because of exposure-measurement uncertainty. Furthermore, the null findings do not rule out a true association between dietary arsenic exposure and breast cancer risk. Another comparative study among women with the family history of the disease reported a significant association between whole blood arsenic and breast cancer risk, with a hazard ratio of 13.2 (95% CI: 4.78–37) [14]. In a prospective study of female teachers conducted in California, results differed from those of the present study, as estimated rice intake using FFQ was associated with a significantly increased risk of breast cancer (HR: 1.07; 95% CI: 1.00-1.15) [41]. Some studies on arsenic in drinking water and others on airborne reported significant associations [42–44], while separate studies on arsenic in airborne and toe nails derivatives found no significant associations [45, 46]. Notably, most studies showing significant effects were conducted in populations with high arsenic exposure, in contrast to the present study, which was in a low-exposure setting.

Arsenic is commonly categorized into two broad forms: organic and inorganic, which differ markedly in toxicity and biological behavior [8]. The inorganic form is considered more toxic and harmful, as it lacks stabilizing carbon bonds. This form occurs in oxidative states relevant to human exposure, including trivalent and pentavalent arsenates [47, 48]. Organic arsenic, in contrast, is generally regarded as less toxic and is often associated with carbon-containing groups derived from sugars, lipids, or cyclic structures [11]. This form comprises multiple species, including methylated compounds such as MMA and DMA, as well as complex compounds, including arsenobetaine, arsenocholice, arsenosugars, and arsenolipids [49]. Inorganic arsenic is predominantly found in rice and cereals, whereas organic arsenic is more commonly present in seafoods [7, 50]. These differences highlight the need to consider both organic and inorganic arsenic food sources in risk assessment, given their differing toxicity levels.

Mechanistically, arsenic exposure may contribute to breast cancer through DNA damage, epigenetic changes, oxidative stress, endocrine disruption, processes that are typically triggered by high doses of arsenic [51–53]. Since this study involved a healthy population with low-level exposure, these carcinogenic pathways may not have been sufficiently activated, which could explain our findings [54, 55]. In addition, dietary arsenic intake often involves organic arsenic compounds, which are less biologically harmful and rapidly excreted [56]. Arsenic-induced carcinogenesis has been linked to skin, lung and bladder cancers at high concentrations [3], whereas evidence for breast cancer remains inconsistent, suggesting that breast tissue may be less susceptible to low-level arsenic exposure. These observations may account for the lack of association observed in this study between dietary arsenic intake and breast cancer.

The large sample size and well-characterized prospective cohort, together with standardized data collection, allowed for one of the first analyses to stratify the association between dietary arsenic intake and serum arsenic by sex, providing insight into potential differences in arsenic metabolism or dietary exposure patterns between male and female participants. Dietary arsenic intake was estimated using detailed dietary information collected through a validated FFQ, enabling a more nuanced assessment of arsenic exposure. The use of high-quality biomonitoring based on archived blood samples was another strength, as blood biomarkers reflect the internal burden of arsenic exposure.

There are several sources of uncertainty in the estimation of concentration data, which may have resulted in both over and underestimation of exposure levels. These include substitution of missing values using similar foods or small sample sets (*n* < 20), and the aggregation of similar foods into broader categories (e.g., sausages). For composite dishes, concentrations were based on the main ingredient, which may not fully capture recipe variability. Estimated iAs intake was derived from tAs intake measurements rather than being directly quantified, potentially introducing uncertainty in the estimation of exposure. Despite adjustment for relevant covariates, residual confounding cannot be ruled out. Dietary factors that may influence oxidative stress, including nutrient intake, overall dietary quality, and dietary patterns, were not adjusted for. These factors could have modified the observed association and therefore represent a potential source of residual confounding.

Additional uncertainty arises from the handling of values below the limit of detection (LOD) or limit of quantification (LOQ), particularly where a substantial proportion of measurements were unquantified. In such cases (< 50% quantified values), the 50th percentile (p50) was used as a proxy threshold, which may have introduced further imprecision. Combining analytical data collected over extended time periods and generated using different analytical methods may have introduced heterogeneity across measurements. The multiple comparisons between dietary arsenic intake and the various subgroups were exploratory and therefore introduced the possibility of chance findings. A substantial measurement error might have occurred in the dietary assessment due to the use of FFQ data and food monitoring databases. This could be due to differences in arsenic concentration among the food sources, cooking and food preparation methods, temporal changes in dietary habits, and agricultural practices, which may have resulted in variation in the actual arsenic content of food. Concentration adjustment factors reflecting differences in ingredient composition were not consistently available or applied, and some items without concentration data could not be included, leading to potential exposure misclassification. In addition, some participants may have been incorrectly classified as having higher or lower dietary arsenic exposure. FFQ reflects what people consumed over a short period, whereas breast cancer is associated with arsenic exposure accumulated over many years. Data on exposure from occupation, industry, and agricultural sources were unavailable. If this misclassification was non-differential with respect to breast cancer, it could have attenuated the observed association toward the null, resulting in an underestimation of the true effect of dietary arsenic intake exposure on breast cancer risk. However, this is unlikely to affect the present analysis, as dietary intake data were collected prospectively within the cohort, before disease onset and independently of health outcomes. In addition, validated FFQ were used. Blood arsenic reflects only recent exposure, so the results should be interpreted with caution. Arsenic speciation was not performed to distinguish the toxicologically more relevant iAs from organic forms in relation to breast cancer. Since these species differ in terms of metabolism, biological activity, and potential toxicity, tAs may not fully reflect the biologically relevant arsenic exposure. The absence of speciation also prevented us from assessing individual arsenic methylation capacity and determining whether specific species contributed differently to the observed associations. A temporal mismatch between exposure assessment and biomarker measurement may also have occurred: serum arsenic primarily reflects recent intake due to high intra-individual variability, whereas dietary intake estimated via the FFQ represents habitual intake over the preceding 12 months. This discrepancy between long-term dietary patterns and short-term blood-based biomarkers may have attenuated the observed correlations.

## Conclusion

Dietary tAs intake was associated with serum arsenic levels in both men and women, whereas the association with estimated iAs intake was observed only in women; neither measure was associated with breast cancer. The lack of association between dietary arsenic intake exposure and breast cancer should be interpreted cautiously. The null findings may reflect relatively low exposure levels, limited exposure contrast, and exposure measurement. Given the generally lower arsenic exposure in European countries than in highly exposed regions such as Asia, these findings may not be generalizable to populations with substantially higher exposure. Future research should incorporate blood speciation analyses to differentiate toxicologically relevant iAs from largely non-toxic organic forms. Prospective longitudinal studies that better align dietary assessment with biomarker measurements are needed to clarify temporal relationships, particularly considering the differing time frames captured by habitual dietary intake and short-term biomarkers. Studies in populations with varying dietary patterns and environmental exposure levels will be important to assess the generalizability of these findings. Additionally, further investigation into biological determinants of arsenic metabolism and biomarker concentrations, including genetic polymorphisms or sex, is warranted.

## Supplementary Information

Below is the link to the electronic supplementary material.

Supplementary Material 1

Supplementary Material 2

## Data Availability

The data supporting the findings of this study will be made available upon reasonable request to the corresponding author.
