# Supplementary material for: Linking dietary-derived arsenic exposure to blood serum arsenic burden, potential food sources, and breast cancer risk in the EPIC-Heidelberg cohort
Source: BMC Cancer. 2026 Nov 12;26:1406. doi: 10.1186/s12885-026-17282-7 (PMC13757149; doi:10.1186/s12885-026-17282-7)
Supplement: Supplementary file 1 [file 12885_2026_17282_MOESM1_ESM.docx]

**Linking Dietary-Derived Arsenic Exposure to Blood Serum Arsenic Burden, Potential Food Sources, and Breast Cancer Risk in the EPIC-Heidelberg Cohort**

Maureen Kipkoech ^1, 2*^ Celine M Beck ^1,3^, Anna Jaeger ^4^, Katrin Blume^4^, Jan Lubiński ^5,6^, Wojciech Marciniak ^5,6^, Róża Derkacz ^5,6^, Theron Johnson ^1^, Rudolf Kaaks ^1^ and Verena A Katzke ^1*^

1. *Division of Cancer Epidemiology, German Cancer Research Center (DKFZ), Im Neuenheimer Feld, Heidelberg, 69120, Baden Württemberg, Germany.
2. Medical Faculty Heidelberg, Heidelberg University, Im NeuenheimerFeld, Heidelberg, 69120, Baden-W ̈urttemberg, Germany.
3. Faculty of Medicine, Ludwig Maximilian University of Munich (LMU Munich), Pettenkoferstraße 8a, Munich, 80336, Bavaria, Germany.
4. Department Exposure, German Federal Institute for Risk Assessment (BfR), Max-Dohrn-Straße 8–10, Berlin, 10589, State, Germany.
5. Department of Genetics and Pathology, Pomeranian Medical University, Powsta ́nc ́ow Wlkp. 72, Szczecin, 70-204, West Pomeranian, Voivodeship, Poland.
6. Read-Gene SA, , ul. Alabastrowa 8, Grzepnica, 72-003, West Pomeranian Voivodeship, Poland.

*Corresponding author(s). E-mail(s): maureen.kipkoech@dkfz.de (ORCID: <https://orcid.org/0000-0002-4224-3671>); v.katzke@dkfz.de (ORCID: <https://orcid.org/0000-0002-6509-6555>)

**Supplementary Table S1.** Food groups and subgroups in EPIC-Heidelberg, translated and adapted by Voss et al (1998)

| Food Groups | Subgroups |
| --- | --- |
| Potatoes and other tubers | Potatoes, other tubers |
| Vegetables | Leaf vegetables, fruit vegetables, root vegetables, cabbage types, mushrooms, legumes and shoots, onions and garlic, stalk vegetables and sprouts, mixed vegetables and vegetable mixes |
| Legumes | Legumes |
| Fruits | Fruit, nuts and seeds, mixed fruits, olives |
| Dairy products | Milk, dairy drinks, yogurt, quark, cheese, desserts made from dairy, cream and coffee cream |
| Cereals and cereal Products | Flour/flakes/starch/grits, pasta/rice/other cereals, bread/crackers/crispbread, breakfast cereals, snack baked goods, doughs |
| Meat and meat products | Beef, veal, pork, mutton/lamb, game, goat, poultry (chicken, turkey, duck, goose), rabbit, other meat, mixed meats, sausages, cold cuts |
| Fish and shellfish | Fish, shellfish/mollusks, fish products |
| Eggs and egg products | Eggs, egg products |
| Fats | Vegetable oils, butter, margarine, deep-frying fat, fish oil, other animal fats |
| Sugar and sweets | Sugar/jam/marmalade, chocolate/bars/confectionery, sweetened or diet chocolate, syrup, ice cream |
| Cakes | Cakes/pastries/pudding with or without dairy, fruit and vegetable desserts |
| Non-alcoholic beverages | Soft drinks/concentrated syrup, coffee, tea/herbal teas, coffee substitutes, water |
| Alcoholic beverages | Wine, sparkling wine, beer, spirits, liqueurs, cocktails/punches |
| Condiments and sauces | Sauces, salad dressings, mayonnaise, dessert sauces, yeast, spices/herbs/aromatics |
| Soups and bouillon | Soups, bouillon |
| Miscellaneous | Soy products, dietetic products, sweeteners, snacks |

**Supplementary Figure S1.** Histogram of inorganic arsenic intake, stratified by sex before logarithmic transformation

**Supplementary Figure S2.** Histogram of log2-transformed inorganic arsenic intake, stratified by sex

**Supplementary Figure S3.** Histogram of total arsenic intake, stratified by sex before logarithmic transformation

**Supplementary Figure S4.** Histogram of log2-transformed total arsenic intake, stratified by sex

**Supplementary Figure S5.** Histogram of serum arsenic, stratified by sex before logarithmic transformation

**Supplementary Figure S6.** Histogram of log2-transformed serum arsenic, stratified by sex

**Supplementary Table S2.** Sex-stratified distribution of dietary arsenic levels (µg/kg bw/d) by food type

| Food Group | tAs-Males | | tAs-Females | | iAs-Males | | iAs-Females | |
| --- | --- | --- | --- | --- | --- | --- | --- | --- |
|  | Min-Max | Median (IQR) | Min-Max | Median (IQR) | Min-Max | Median (IQR) | Min-Max | Median (IQR) |
| Potatoes | 6e-04–0.0596 | 0.01 (0.01) | 0–0.0984 | 0.01 (0.01) | 4e-04–0.0416 | 0.01 (0) | 0–0.0689 | 0.01 (0) |
| Leafy Vegetables (Except Cabbages) | 0–0.0187 | 0 (0) | 0–0.0463 | 0 (0) | 0–0.0131 | 0 (0) | 0–0.0324 | 0 (0) |
| Fruiting Vegetables | 0.0 - 0.0283 | 0 (0) | 3e-04–0.0652 | 0 (0) | 0–0.0199 | 0 (0) | 2e-04–0.0455 | 0 (0) |
| Root Vegetables | 0.0 - 0.0161 | 0 (0) | 0–0.0349 | 0 (0) | 0–0.0112 | 0 (0) | 0–0.0243 | 0 (0) |
| Cabbages | 0.0 - 0.0137 | 0 (0) | 0–0.0259 | 0 (0) | 0–0.0096 | 0 (0) | 0–0.0182 | 0 (0) |
| Mushrooms | 0.0 - 0.0081 | 0 (0) | 0–0.0047 | 0 (0) | 0–0.0057 | 0 (0) | 0–0.0033 | 0 (0) |
| Grain And Pod Vegetables | 0.0 - 0.0079 | 0 (0) | 0–0.0042 | 0 (0) | 0–0.0055 | 0 (0) | 0–0.003 | 0 (0) |
| Onion, Garlic | 0.0 - 0.0049 | 0 (0) | 0–0.005 | 0 (0) | 0–0.0035 | 0 (0) | 0–0.0035 | 0 (0) |
| Stalk Vegetables, Sprouts | 0.0 - 0–0.0134 | 0 (0) | 0–0.0277 | 0 (0) | 0–0.0094 | 0 (0) | 0–0.0194 | 0 (0) |
| Mixed Salad, Mixed Vegetables | 0–0.0113 | 0 (0) | 0–0.0106 | 0 (0) | 0–0.0079 | 0 (0) | 0–0.0074 | 0 (0) |
| Legumes | 0–0.0191 | 0 (0) | 0–0.0275 | 0 (0) | 0–0.0134 | 0 (0) | 0–0.0192 | 0 (0) |
| Fruits | 0–0.0688 | 0.01 (0.01) | 0–0.0762 | 0.01 (0.01) | 0–0.0482 | 0 (0) | 0–0.0533 | 0.01 (0.01) |
| Nuts, Seeds, Nut Spread | 0–0.0227 | 0 (0) | 0–0.04 | 0 (0) | 0–0.0159 | 0 (0) | 0–0.028 | 0 (0) |
| Mixed Fruits | 0–0.0054 | 0 (0) | 0–0.0093 | 0 (0) | 0–0.0037 | 0 (0) | 0–0.0064 | 0 (0) |
| Milk | 0–0.2298 | 0 (0.01) | 0–0.2194 | 0.01 (0.02) | 0–0.1609 | 0 (0.01) | 0–0.1536 | 0.01 (0.01) |
| Milk Beverages | 0–0.2089 | 0 (0) | 0–0.2309 | 0 (0) | 0–0.1463 | 0 (0) | 0–0.1616 | 0 (0) |
| Yogurt | 0–0.3811 | 0.01 (0.02) | 0–0.4007 | 0.01 (0.03) | 0–0.2668 | 0 (0.01) | 0–0.2805 | 0.01 (0.02) |
| Fromage Blanc, Petits Suisses | 0–0.0379 | 0 (0) | 0–0.0607 | 0 (0) | 0–0.0265 | 0 (0) | 0–0.0425 | 0 (0) |
| Cheeses (Including Fresh Cheeses) | 0–0.047 | 0 (0) | 0–0.0584 | 0.01 (0.01) | 0–0.0328 | 0 (0) | 0–0.0409 | 0 (0) |
| Cream Desserts, Puddings (Milk Based) | 0–0.0307 | 0 (0) | 0–0.0287 | 0 (0) | 0–0.0214 | 0 (0) | 0–0.0201 | 0 (0) |
| Dairy Creams | 0–0.0062 | 0 (0) | 0–0.0134 | 0 (0) | 0–0.0044 | 0 (0) | 0–0.0094 | 0 (0) |
| Flour, Flakes, Starches, Semolina | 0–0.0096 | 0 (0) | 0–0.0093 | 0 (0) | 0–0.0067 | 0 (0) | 0–0.0065 | 0 (0) |
| Rice | 0–0.3464 | 0.02 (0.02) | 0–0.4117 | 0.02 (0.02) | 0–0.2425 | 0.01 (0.01) | 0–0.2882 | 0.01 (0.01) |
| Pasta, Other Grain | 0–0.0576 | 0.01 (0) | 0–0.0479 | 0.01 (0.01) | 0–0.0403 | 0 (0) | 0–0.0336 | 0 (0) |
| Bread | 0–0.0965 | 0.02 (0.01) | 0–0.187 | 0.02 (0.01) | 0–0.0675 | 0.01 (0.01) | 0–0.1308 | 0.01 (0.01) |
| Crispbread, Rusks | 0–0.0191 | 0 (0) | 0–0.0308 | 0 (0) | 0–0.0134 | 0 (0) | 0–0.0215 | 0 (0) |
| Breakfast Cereals | 0–0.0308 | 0 (0) | 0–0.0549 | 0 (0) | 0–0.0216 | 0 (0) | 0–0.0384 | 0 (0) |
| Salty Biscuits, Aperitif Biscuits | 0–0.0235 | 0 (0) | 0–0.0159 | 0 (0) | 0–0.0165 | 0 (0) | 0–0.0111 | 0 (0) |
| Dough And Pastry | 0–0.0051 | 0 (0) | 0–0.01 | 0 (0) | 0–0.0035 | 0 (0) | 0–0.007 | 0 (0) |
| Fresh Meat Unclassified | 0–8e-04 | 0 (0) | 0–7e-04 | 0 (0) | 0–6e-04 | 0 (0) | 0–5e-04 | 0 (0) |
| Beef | 0–0.0201 | 0 (0) | 0–0.0205 | 0 (0) | 0–0.0141 | 0 (0) | 0–0.0144 | 0 (0) |
| Veal | 0–0.005 | 0 (0) | 0–0.0031 | 0 (0) | 0–0.0035 | 0 (0) | 0–0.0022 | 0 (0) |
| Pork | 0–0.0336 | 0 (0) | 0–0.018 | 0 (0) | 0–0.0236 | 0 (0) | 0–0.0125 | 0 (0) |
| Mutton/Lamb | 0–0.0061 | 0 (0) | 0–0.0041 | 0 (0) | 0–0.0042 | 0 (0) | 0–0.0029 | 0 (0) |
| Unclassified And Other Poultry | 0–0.0147 | 0 (0) | 0–0.0476 | 0 (0) | 0–0.0103 | 0 (0) | 0–0.0333 | 0 (0) |
| Chicken, Hen | 0–0.0152 | 0 (0) | 0–0.0147 | 0 (0) | 0–0.0106 | 0 (0) | 0–0.0103 | 0 (0) |
| Rabbit (Domestic) | 0–4e-04 | 0 (0) | 0–3e-04 | 0 (0) | 0–3e-04 | 0 (0) | 0–2e-04 | 0 (0) |
| Processed Meat | 0–0.0562 | 0.01 (0.01) | 0–0.0398 | 0.01 (0.01) | 0–0.0394 | 0.01 (0) | 0–0.0278 | 0 (0) |
| Offals | 0–0.0051 | 0 (0) | 0–0.0064 | 0 (0) | 0–0.0035 | 0 (0) | 0–0.0045 | 0 (0) |
| Fish | 0–1.7664 | 0.23 (0.28) | 0–8.8054 | 0.22 (0.26) | 0–0.0353 | 0 (0.01) | 0–0.1761 | 0 (0.01) |
| Fish Products, Fish in Crumbs | 0–0.9794 | 0.06 (0.07) | 0–6.2775 | 0.05 (0.06) | 0–0.0196 | 0 (0) | 0–0.1256 | 0 (0) |
| Egg | 0–0.0101 | 0 (0) | 0–0.0114 | 0 (0) | 0–0.0071 | 0 (0) | 0–0.0079 | 0 (0) |
| Unclassified | 0–0.0015 | 0 (0) | 0–0.0013 | 0 (0) | 0–0.001 | 0 (0) | 0–8e-04 | 0 (0) |
| Vegetable Oils | 0–0.0151 | 0 (0) | 0–0.0161 | 0 (0) | 0–0.0106 | 0 (0) | 0–0.0113 | 0 (0) |
| Butter | 0–0.018 | 0 (0) | 0–0.0129 | 0 (0) | 0–0.0125 | 0 (0) | 0–0.0091 | 0 (0) |
| Margarines | 0–0.0123 | 0 (0) | 0–0.0139 | 0 (0) | 0–0.0085 | 0 (0) | 0–0.0097 | 0 (0) |
| Sugar, Honey, Jam | 0–0.0728 | 0 (0) | 0–0.0722 | 0 (0) | 0–0.051 | 0 (0) | 0–0.0506 | 0 (0) |
| Chocolate, Candy Bars, Paste | 0–0.0849 | 0 (0) | 0–0.1024 | 0 (0) | 0–0.0595 | 0 (0) | 0–0.0717 | 0 (0) |
| Confectionery Non-Chocolate | 0–0.0048 | 0 (0) | 0–0.0086 | 0 (0) | 0–0.0033 | 0 (0) | 0–0.006 | 0 (0) |
| Ice Cream, Water Ice | 0–0.0087 | 0 (0) | 0–0.013 | 0 (0) | 0–0.0061 | 0 (0) | 0–0.0091 | 0 (0) |
| Ice Cream | 0–0.0017 | 0 (0) | 0–0.0023 | 0 (0) | 0–0.0012 | 0 (0) | 0–0.0016 | 0 (0) |
| Cakes, Pies, Pastries | 0–0.0775 | 0 (0.01) | 0–0.1131 | 0.01 (0.01) | 0–0.0543 | 0 (0) | 0–0.0792 | 0 (0) |
| Unclassified | 0–0.2699 | 0 (0) | 0–0.0949 | 0 (0) | 0–0.1889 | 0 (0) | 0–0.0665 | 0 (0) |
| Fruit And Vegetable Juices | 0–0.2214 | 0.01 (0.02) | 0–0.1853 | 0.01 (0.02) | 0–0.1549 | 0.01 (0.01) | 0–0.1297 | 0.01 (0.01) |
| Carbonated, Soft, Isotonic | 0–0.1481 | 0 (0) | 0–0.1506 | 0 (0) | 0–0.1037 | 0 (0) | 0–0.1054 | 0 (0) |
| Coffee | 0–0.4603 | 0.04 (0.05) | 0–0.6014 | 0.05 (0.06) | 0–0.3222 | 0.03 (0.04) | 0–0.421 | 0.03 (0.04) |
| Tea | 0–0.0705 | 0 (0.01) | 0–0.097 | 0 (0.01) | 0–0.0493 | 0 (0) | 0–0.0679 | 0 (0) |
| Herbal Tea | 0–0.0707 | 0 (0) | 0–0.1086 | 0 (0.01) | 0–0.0495 | 0 (0) | 0–0.076 | 0 (0.01) |
| Chicory, Substitutes | 0–0.0165 | 0 (0) | 0–0.034 | 0 (0) | 0–0.0116 | 0 (0) | 0–0.0238 | 0 (0) |
| Waters | 0–0.0382 | 0 (0.01) | 0–0.07 | 0.01 (0.01) | 0–0.0382 | 0 (0.01) | 0–0.07 | 0.01 (0.01) |
| Wine | 0–0.3896 | 0.01 (0.04) | 0–0.315 | 0.01 (0.03) | 0–0.2727 | 0.01 (0.03) | 0–0.2205 | 0.01 (0.02) |
| Beer, Cider | 0–0.4488 | 0.02 (0.03) | 0–0.2067 | 0 (0.01) | 0–0.3142 | 0.01 (0.02) | 0–0.1447 | 0 (0) |
| Unclassified And Other Sauces | 0–0.0073 | 0 (0) | 0–0.0067 | 0 (0) | 0–0.0051 | 0 (0) | 0–0.0047 | 0 (0) |
| Tomato Sauces | 0–0.004 | 0 (0) | 0–0.0074 | 0 (0) | 0–0.0029 | 0 (0) | 0–0.0052 | 0 (0) |
| Dressing Sauces | 0–0.0011 | 0 (0) | 0–6e-04 | 0 (0) | 0–8e-04 | 0 (0) | 0–4e-04 | 0 (0) |
| Spices, Herbs and Flavorings | 0–6e-04 | 0 (0) | 0–9e-04 | 0 (0) | 0–4e-04 | 0 (0) | 0–6e-04 | 0 (0) |
| Condiments | 0–4e-04 | 0 (0) | 0–7e-04 | 0 (0) | 0–3e-04 | 0 (0) | 0–5e-04 | 0 (0) |
| Soups | 0–0.0341 | 0 (0) | 0–0.0756 | 0 (0) | 0–0.0239 | 0 (0) | 0–0.0529 | 0 (0) |
| Bouillon | 0–0.0033 | 0 (0) | 0–0.0041 | 0 (0) | 0–0.0023 | 0 (0) | 0–0.0028 | 0 (0) |
| Unclassified | 0–0.0139 | 0 (0) | 0–0.0292 | 0 (0) | 0–0.0098 | 0 (0) | 0–0.0205 | 0 (0) |
| Soya Products | 0–0.001 | 0 (0) | 0–0.002 | 0 (0) | 0–7e-04 | 0 (0) | 0–0.0014 | 0 (0) |

**Supplementary Figure S7**. Median inorganic arsenic intake across 17 food groups, stratified by sex

**Supplementary Table S3.** Correlation between total serum arsenic levels (µg/L) and dietary arsenic levels (µg/kg bw/d)

| Sex | Type of arsenic | Spearman coefficient^a^ | P value |
| --- | --- | --- | --- |
| Overall | tAs | 0.20 | <0.0001 |
| Overall | iAs | 0.07 | 0.00033 |
| Male | tAs | 0.20 | <0.0001 |
| Female | tAs | 0.21 | <0.0001 |
| Male | iAs | 0.07 | 0.02 |
| Female | iAs | 0.07 | 0.01 |

^a^ Adjusted for sex and age at recruitment

**Supplementary Table S4.** Correlation between total serum arsenic concentrations (µg/L) and dietary arsenic intake (µg/kg body weight/day) across 17 food groups and their subgroups

| Food GroupS | | Total Arsenic | | Inorganic Arsenic | |
| --- | --- | --- | --- | --- | --- |
|  |  | ρ | p value | ρ^a^ | p value |
| 01 | Potatoes and tubers | 0.019 | 0.3226 | 0.019 | 0.3141 |
| 02 | Vegetables | 0.052 | 0.0074* | 0.051 | 0.0082* |
|  | Leafy vegetables (except cabbages) | 0.031 | 0.1129 | 0.033 | 0.0872 |
|  | Fruiting vegetables | 0.050 | 0.0094 | 0.049 | 0.0122 |
|  | Root vegetables | 0.002 | 0.9345 | 0.005 | 0.8161 |
|  | Cabbages | 0.019 | 0.3174 | 0.020 | 0.3013 |
|  | Mushrooms | 0.024 | 0.2497 | 0.028 | 0.1918 |
|  | Grain and pod vegetables | 0.012 | 0.5678 | 0.008 | 0.7351 |
|  | Onion, garlic | 0.067 | 0.0006 | 0.056 | 0.0049 |
|  | Stalk vegetables, sprouts | -0.002 | 0.9025 | -0.008 | 0.7062 |
|  | Mixed salad, mixed vegetables | 0.013 | 0.5046 | 0.007 | 0.7258 |
| 03 | Legumes | 0.001 | 0.9533 | -0.002 | 0.9171 |
| 04 | Fruits, nuts, seeds | 0.002 | 0.9149 | 0.003 | 0.8853 |
| 05 | Dairy products | -0.020 | 0.3025 | -0.020 | 0.3021 |
| 06 | Cereal and cereal products | -0.001 | 0.9422 | -0.001 | 0.9459 |
| 07 | Meat and meat products | 0.010 | 0.5893 | 0.012 | 0.5397 |
| 08 | Fish and shellfish | 0.211 | <0.0001* | 0.211 | <0.0001* |
|  | 0801 Fish | 0.209 | <0.0001* | 0.208 | <0.0001* |
|  | 0803 Fish products, fish in crumbs | 0.210 | <0.0001* | 0.210 | <0.0001* |
| 09 | Eggs and egg products | 0.032 | 0.1146 | 0.042 | 0.0535 |
| 10 | Fat | 0.027 | 0.1574 | 0.026 | 0.1784 |
| 11 | Sugar and confectionary | -0.032 | 0.1027 | -0.032 | 0.1050 |
| 12 | Cakes and biscuits | 0.004 | 0.8214 | 0.005 | 0.7948 |
| 13 | Non-alcoholic beverages | 0.013 | 0.5146 | 0.015 | 0.4318 |
| 14 | Alcoholic beverages | 0.125 | <0.0001* | 0.125 | <0.0001* |
|  | 1401 Wine | 0.110 | <0.0001* | 0.110 | <0.0001* |
|  | 1403 Beer, cider | 0.086 | 0.0001* | 0.086 | 0.0001* |
| 15 | Condiments and sauces | -0.040 | 0.0419* | -0.034 | 0.0828 |
| 16 | Soups, bouillons | 0.023 | 0.2364 | 0.025 | 0.2128 |
| 17 | Miscellaneous | 0.042 | 0.1738 | 0.033 | 0.2801 |

^a^ρ = Spearman`s rank correlation coefficient

**Supplementary Table S5.** Sensitivity analysis for the association between dietary arsenic intake (Median lower Bound) and breast cancer in the EPIC-Heidelberg case–cohort (HR and 95%CI).

|  | Quartile of Arsenic | | | | *p*-Trend |
| --- | --- | --- | --- | --- | --- |
| Dietary Arsenic | Q1 | Q2 | Q3 | Q4 |  |
| Total dietary arsenic |  |  |  |  |  |
| Number of cases | 149 | 171 | 140 | 171 |  |
| Sub-cohort non-cases | 422 | 431 | 429 | 426 |  |
| Median arsenic (µg/kg bw/d) | 0.08 | 0.22 | 0.37 | 0.61 |  |
| Crude HR (95% CI) | Ref | 1.09 (0.92–1.3) | 1.03 (0.87–1.23) | 1.14 (0.96–1.36) | 0.25 |
| Adjusted HR (95% CI)^a^ | Ref | 1.07 (0.9–1.28) | 1.03 (0.87–1.22) | 1.14 (0.96–1.35) | 0.34 |
| Inorganic dietary arsenic |  |  |  |  |  |
| Number of cases | 161 | 156 | 136 | 178 |  |
| Sub-cohort non-cases | 425 | 422 | 430 | 431 |  |
| Median arsenic (µg/kg bw/d) | 0.01 | 0.02 | 0.03 | 0.05 |  |
| Crude HR (95% CI) | Ref | 1.1 (0.93–1.3) | 1.18 (1–1.4) | 0.15 (0.96–1.37) | 0.12 |
| Adjusted HR (95% CI)^a^ | Ref | 1.05 (0.89–1.26) | 1.16 (0.98–1.38) | 0.14 (0.95–1.36) | 0.32 |

^a^ Adjusted for level of education, BMI, smoking status, alcohol use, physical activity, parity status, use of oral contraceptives/HRT, breastfeeding history, age at menarche, and family history of breast cancer.

**Supplementary Table S6**. Sensitivity analysis for the association between dietary arsenic levels (Median Upper Bound) and breast cancer risk by menopausal status at baseline (HR and 95%CI).

|  | Quartile of Arsenic | | | | *p*-Trend |
| --- | --- | --- | --- | --- | --- |
| Menopausal status at recruitment | Q1 | Q2 | Q3 | Q4 |  |
| Premenopausal: tAs |  |  |  |  |  |
| Number of cases | 38 | 56 | 54 | 63 |  |
| Sub-cohort non-cases | 147 | 146 | 148 | 145 |  |
| Median arsenic (µg/kg bw/d) | 0.34 | 0.50 | 0.68 | 0.96 |  |
| Crude HR (95% CI) | Ref | 1.37 (1.02–1.85) | 1.04 (0.77–1.4) | 1.13 (0.84–1.51) | 0.03 |
| Adjusted HR (95% CI)^a^ | Ref | 1.29 (0.93–1.78) | 1.06 (0.79–1.43) | 1.1 (0.81–1.48) | 0.09 |
| Postmenopausal: tAs |  |  |  |  |  |
| Number of cases | 82 | 78 | 74 | 75 |  |
| Sub-cohort non-cases | 208 | 218 | 215 | 216 |  |
| Median arsenic (µg/kg bw/d) | 0.32 | 0.49 | 0.67 | 0.96 |  |
| Crude HR (95% CI) | Ref | 0.92 (0.72–1.17) | 1.03 (0.8–1.31) | 1.02 (0.8–1.3) | 0.03 |
| Adjusted HR (95% CI)^a^ | Ref | 0.86 (0.67–1.12) | 1.02 (0.8–1.3) | 1.02 (0.79–1.3) | 0.10 |
| Premenopausal: iAs |  |  |  |  |  |
| Number of cases | 47 | 44 | 66 | 54 |  |
| Sub-cohort non-cases | 148 | 149 | 145 | 144 |  |
| Median arsenic (µg/kg bw/d) | 0.17 | 0.22 | 0.27 | 0.34 |  |
| Crude HR (95% CI) | Ref | 1.1 (0.81–1.48) | 0.92 (0.68–1.24) | 0.77 (0.58–1.03) | 0.62 |
| Adjusted HR (95% CI)^a^ | Ref | 0.96 (0.67–1.38) | 0.94 (0.7–1.27) | 0.8 (0.6–1.07) | 0.77 |
| Postmenopausal: iAs |  |  |  |  |  |
| Number of cases | 67 | 89 | 80 | 73 |  |
| Sub-cohort non-cases | 218 | 207 | 215 | 217 |  |
| Median arsenic (µg/kg bw/d) | 0.15 | 0.20 | 0.24 | 0.32 |  |
| Crude HR (95% CI) | Ref | 0.96 (0.74–1.24) | 0.88 (0.69–1.13) | 1.09 (0.86–1.38) | 0.62 |
| Adjusted HR (95% CI)^a^ | Ref | 0.87 (0.64–1.18) | 0.92 (0.72–1.18) | 1.05 (0.83–1.33) | 0.77 |

^a^ Covariates included in the models were consistent with Model 5 unless otherwise specified. HRs (95% CIs) were estimated using Cox proportional hazards regression models.

.

**Supplementary Table S7**. Sensitivity analysis for the association between dietary arsenic levels (Median Upper Bound) and breast cancer risk by menopausal status at diagnosis (HR and 95%CI).

|  | Quartile of Arsenic | | | | *p*-Trend |
| --- | --- | --- | --- | --- | --- |
| Menopausal status at diagnosis | Q1 | Q2 | Q3 | Q4 |  |
| Premenopausal: tAs |  |  |  |  |  |
| Number of cases | 15 | 18 | 22 | 34 |  |
| Sub-cohort non-cases | 422 | 432 | 430 | 424 |  |
| Median arsenic (µg/kg bw/d) | 0.33 | 0.50 | 0.68 | 0.97 |  |
| Crude HR (95% CI) | Ref | 1.24 (0.91–1.69) | 1.34 (0.97–1.86) | 0.95 (0.67–1.34) | 0.1 |
| Adjusted HR (95% CI)^a^ | Ref | 1.2 (0.86–1.67) | 1.37 (0.99–1.91) | 0.93 (0.66–1.31) | 0.16 |
| Postmenopausal: tAs |  |  |  |  |  |
| Number of cases | 130 | 148 | 130 | 134 |  |
| Sub-cohort non-cases | 420 | 431 | 429 | 425 |  |
| Median arsenic (µg/kg bw/d) | 0.33 | 0.50 | 0.67 | 0.96 |  |
| Crude HR (95% CI) | Ref | 1.02 (0.85–1.22) | 0.97 (0.81–1.17) | 1.1 (0.92–1.32) | 0.86 |
| Adjusted HR (95% CI)^a^ | Ref | 1 (0.82–1.21) | 0.97 (0.81–1.16) | 1.1 (0.92–1.32) | 0.97 |
| Premenopausal: iAs |  |  |  |  |  |
| Number of cases | 14 | 11 | 33 | 31 |  |
| Sub-cohort non-cases | 431 | 425 | 426 | 426 |  |
| Median arsenic (µg/kg bw/d) | 0.15 | 0.20 | 0.25 | 0.33 |  |
| Crude HR (95% CI) | Ref | 1.08 (0.78–1.51) | 1.03 (0.74–1.42) | 0.87 (0.63–1.21) | 0.79 |
| Adjusted HR (95% CI)^a^ | Ref | 1.01 (0.68–1.5) | 1.03 (0.74–1.43) | 0.87 (0.63–1.21) | 0.97 |
| Postmenopausal: iAs |  |  |  |  |  |
| Number of cases | 126 | 145 | 141 | 130 |  |
| Sub-cohort non-cases | 430 | 422 | 427 | 429 |  |
| Median arsenic (µg/kg bw/d) | 0.15 | 0.20 | 0.25 | 0.33 |  |
| Crude HR (95% CI) | Ref | 1.03 (0.86–1.24) | 0.95 (0.79–1.14) | 1.01 (0.84–1.21) | 0.79 |
| Adjusted HR (95% CI)^a^ | Ref | 1.01 (0.81–1.26) | 0.96 (0.8–1.15) | 0.98 (0.82–1.17) | 0.97 |

^a^ Covariates included in the models were consistent with Model 5 unless otherwise specified. HRs (95% CIs) were estimated using Cox proportional hazards regression models.

**Supplementary Table S8.** Sensitivity analysis for the association between dietary arsenic levels (Mean upper and lower Bound) and breast cancer in the EPIC-Heidelberg case–cohort (HR and 95% CI).

|  | Quartile of Arsenic | | | | *p*-Trend |
| --- | --- | --- | --- | --- | --- |
| Dietary Arsenic Mean Upper and Lower Bound | Q1 | Q2 | Q3 | Q4 |  |
| Mean Upper Bound: tAs |  |  |  |  |  |
| Number of cases | 146 | 162 | 157 | 166 |  |
| Sub-cohort non-cases | 424 | 429 | 431 | 424 |  |
| Median arsenic (µg/kg bw/d) | 0.45 | 0.67 | 0.89 | 1.26 |  |
| Crude HR (95% CI) | Ref | 1.08 (0.91–1.28) | 0.99 (0.83–1.17) | 1.07 (0.9–1.26) | 0.39 |
| Adjusted HR (95% CI)^a^ | Ref | 1.05 (0.88–1.25) | 1 (0.84–1.18) | 1.08 (0.91–1.28) | 0.59 |
| Mean Upper Bound: iAs |  |  |  |  |  |
| Number of cases | 139 | 158 | 175 | 159 |  |
| Sub-cohort non-cases | 431 | 424 | 427 | 426 |  |
| Median arsenic (µg/kg bw/d) | 0.22 | 0.29 | 0.36 | 0.47 |  |
| Crude HR (95% CI) | Ref | 1.03 (0.86–1.22) | 0.91 (0.76–1.08) | 0.94 (0.8–1.11) | 0.88 |
| Adjusted HR (95% CI)^a^ | Ref | 0.97 (0.79–1.18) | 0.92 (0.77–1.09) | 0.93 (0.79–1.11) | 0.62 |
| Mean Lower Bound: tAs |  |  |  |  |  |
| Number of cases | 160 | 159 | 140 | 172 |  |
| Sub-cohort non-cases | 421 | 432 | 430 | 425 |  |
| Median arsenic (µg/kg bw/d) | 0.18 | 0.37 | 0.56 | 0.85 |  |
| Crude HR (95% CI) | Ref | 1.07 (0.9–1.26) | 1.1 (0.93–1.3) | 1.08 (0.91–1.29) | 0.36 |
| Adjusted HR (95% CI)^a^ | Ref | 1.05 (0.88–1.24) | 1.11 (0.93–1.31) | 1.08 (0.91–1.28) | 0.45 |
| Mean Lower Bound: iAs |  |  |  |  |  |
| Number of cases | 156 | 139 | 180 | 156 |  |
| Sub-cohort non-cases | 428 | 422 | 423 | 435 |  |
| Median arsenic (µg/kg bw/d) | 0.06 | 0.09 | 0.11 | 0.16 |  |
| Crude HR (95% CI) | Ref | 1.01 (0.85–1.2) | 0.97 (0.82–1.15) | 0.89 (0.75–1.06) | 0.97 |
| Adjusted HR (95% CI)^a^ | Ref | 0.94 (0.78–1.14) | 0.98 (0.82–1.16) | 0.91 (0.77–1.08) | 0.47 |

^a^ Covariates included in the models were consistent with Model 5 unless otherwise specified. HRs (95% CIs) were estimated using Cox proportional hazards regression models.

**Supplementary Table S9.** Sensitivity analysis for the association between dietary arsenic exposure and all-cause mortality (excluding breast cancer cases) in the EPIC-Heidelberg case–cohort (HR and 95%CI).

|  | Quartile of Arsenic | | | | p-Trend |
| --- | --- | --- | --- | --- | --- |
| Dietary Arsenic | Q1 | Q2 | Q3 | Q4 |  |
| Median Upper Bound: tAs |  |  |  |  |  |
| Number of deaths | 52 | 42 | 47 | 46 |  |
| Sub-cohort non-cases | 412 | 422 | 419 | 421 |  |
| Median arsenic (µg/kg bw/d) | 0.33 | 0.50 | 0.67 | 0.97 |  |
| Crude HR (95% CI) | Ref | 0.89 (0.62–1.28) | 0.94 (0.65–1.34) | 1.01 (0.71–1.45) | 0.51 |
| Adjusted HR (95% CI)^a^ | Ref | 0.91 (0.62–1.34) | 0.95 (0.65–1.37) | 0.95 (0.66–1.36) | 0.61 |
| Median Upper Bound: iAs |  |  |  |  |  |
| Number of deaths | 54 | 56 | 44 | 33 |  |
| Sub-cohort non-cases | 404 | 414 | 426 | 430 |  |
| Median arsenic (µg/kg bw/d) | 0.15 | 0.20 | 0.25 | 0.33 |  |
| Crude HR (95% CI) | Ref | 0.86 (0.57–1.28) | 0.67 (0.46–0.97) | 1.06 (0.75–1.49) | 0.12 |
| Adjusted HR (95% CI)^a^ | Ref | 0.93 (0.59–1.46) | 0.6 (0.41–0.87) | 1.12 (0.79–1.58) | 0.32 |
| Median Lower Bound: tAs |  |  |  |  |  |
| Number of cases | 52 | 45 | 43 | 47 |  |
| Sub-cohort non-cases | 411 | 422 | 420 | 421 |  |
| Median arsenic (µg/kg bw/d) | 0.45 | 0.67 | 0.89 | 1.25 |  |
| Crude HR (95% CI) | Ref | 0.86 (0.6–1.24) | 0.89 (0.62–1.28) | 1.09 (0.76–1.56) | 0.40 |
| Adjusted HR (95% CI)^a^ | Ref | 0.91 (0.62–1.34) | 0.9 (0.62–1.3) | 1.02 (0.71–1.46) | 0.59 |
| Median Lower Bound: iAs |  |  |  |  |  |
| Number of cases | 55 | 52 | 48 | 32 |  |
| Sub-cohort non-cases | 404 | 416 | 423 | 431 |  |
| Median arsenic (µg/kg bw/d) | 0.22 | 0.29 | 0.36 | 0.47 |  |
| Crude HR (95% CI) | Ref | 0.79 (0.53–1.17) | 0.73 (0.5–1.06) | 0.81 (0.57–1.15) | 0.26 |
| Adjusted HR (95% CI)^a^ | Ref | 0.86 (0.55–1.35) | 0.67 (0.46–0.97) | 0.85 (0.6–1.21) | 0.51 |

^a^ Covariates included in the models were consistent with Model 5 unless otherwise specified. HRs (95% CIs) were estimated using Cox proportional hazards regression models.
