# Supplementary material for: Linking dietary-derived arsenic exposure to blood serum arsenic burden, potential food sources, and breast cancer risk in the EPIC-Heidelberg cohort
Source: BMC Cancer. 2026 Nov 12;26:1406. doi: 10.1186/s12885-026-17282-7 (PMC13757149; doi:10.1186/s12885-026-17282-7)
Supplement: Supplementary file 2 [file 12885_2026_17282_MOESM2_ESM.docx]

**Linking Dietary-Derived Arsenic Exposure to Blood Serum Arsenic Burden, Potential Food Sources, and Breast Cancer Risk in the EPIC-Heidelberg Cohort**

Maureen Kipkoech ^1, 2*^ Celine M Beck ^1,3,4,5^, Anna Jaeger ^6^, Katrin Blume^6^, Jan Lubiński ^7,8^, Wojciech Marciniak ^7,8^, Róża Derkacz ^7,8^, Theron Johnson ^1^, Rudolf Kaaks ^1^ and Verena A Katzke ^1*^

1. *Division of Cancer Epidemiology, German Cancer Research Center (DKFZ), Im Neuenheimer Feld, Heidelberg, 69120, Baden Württemberg, Germany.
2. Medical Faculty Heidelberg, Heidelberg University, Im NeuenheimerFeld, Heidelberg, 69120, Baden-W ̈urttemberg, Germany.
3. Institute for Medical Information Processing, Biometry, and Epidemiology, Chair of Public Health and Health Services Research, Faculty of Medicine, LMU Munich, Munich, Germany
4. Pettenkofer School of Public Health, Munich, Germany
5. Institute of Clinical Epidemiology and Biometry, Julius Maximilian University of Würzburg, Würzburg, Germany
6. Department Exposure, German Federal Institute for Risk Assessment (BfR), Max-Dohrn-Straße 8–10, Berlin, 10589, State, Germany.
7. Department of Genetics and Pathology, Pomeranian Medical University, Powsta ́nc ́ow Wlkp. 72, Szczecin, 70-204, West Pomeranian, Voivodeship, Poland.
8. Read-Gene SA, ul. Alabastrowa 8, Grzepnica, 72-003, West Pomeranian Voivodeship, Poland.

*Corresponding author(s). E-mail(s): maureen.kipkoech@dkfz.de (ORCID: https://orcid.org/0000-0002-4224-3671); v.katzke@dkfz.de (ORCID: https://orcid.org/0000-0002-6509-6555)

**Supplementary Table S1.** Food groups and subgroups in EPIC-Heidelberg, translated and adapted by Voss et al (1998)

| Food Groups | Subgroups |
| --- | --- |
| Potatoes and other tubers | Potatoes, other tubers |
| Vegetables | Leaf vegetables, fruit vegetables, root vegetables, cabbage types, mushrooms, legumes and shoots, onions and garlic, stalk vegetables and sprouts, mixed vegetables and vegetable mixes |
| Legumes | Legumes |
| Fruits | Fruit, nuts and seeds, mixed fruits, olives |
| Dairy products | Milk, dairy drinks, yogurt, quark, cheese, desserts made from dairy, cream and coffee cream |
| Cereals and cereal Products | Flour/flakes/starch/grits, pasta/rice/other cereals, bread/crackers/crispbread, breakfast cereals, snack baked goods, doughs |
| Meat and meat products | Beef, veal, pork, mutton/lamb, game, goat, poultry (chicken, turkey, duck, goose), rabbit, other meat, mixed meats, sausages, cold cuts |
| Fish and shellfish | Fish, shellfish/mollusks, fish products |
| Eggs and egg products | Eggs, egg products |
| Fats | Vegetable oils, butter, margarine, deep-frying fat, fish oil, other animal fats |
| Sugar and sweets | Sugar/jam/marmalade, chocolate/bars/confectionery, sweetened or diet chocolate, syrup, ice cream |
| Cakes | Cakes/pastries/pudding with or without dairy, fruit and vegetable desserts |
| Non-alcoholic beverages | Soft drinks/concentrated syrup, coffee, tea/herbal teas, coffee substitutes, water |
| Alcoholic beverages | Wine, sparkling wine, beer, spirits, liqueurs, cocktails/punches |
| Condiments and sauces | Sauces, salad dressings, mayonnaise, dessert sauces, yeast, spices/herbs/aromatics |
| Soups and bouillon | Soups, bouillon |
| Miscellaneous | Soy products, dietetic products, sweeteners, snacks |

**Supplementary Figure S1.** Histogram of estimated inorganic arsenic intake, stratified by sex before logarithmic transformation

**Supplementary Figure S2.** Histogram of log2-transformed Estimated inorganic arsenic intake, stratified by sex

**Supplementary Figure S3.** Histogram of total arsenic intake, stratified by sex before logarithmic transformation

**Supplementary Figure S4.** Histogram of log2-transformed total arsenic intake, stratified by sex

**Supplementary Figure S5.** Histogram of serum arsenic, stratified by sex before logarithmic transformation

**Supplementary Figure S6.** Histogram of log2-transformed serum arsenic, stratified by sex

**Supplementary Table S2.** Percentage contribution of dietary food sources to total and estimated inorganic arsenic intake

| **Food Group** | **Overall-tAs intake** | **Males-tAs intake** | **Females-tAs intake** | **Overall-Estimated iAs intake** | **Males-** **Estimated iAs intake** | **Females-** **Estimated iAs intake** |
| --- | --- | --- | --- | --- | --- | --- |
| Potatoes | 2.1% (2.0–2.2%) | 2.1% (2.0–2.2%) | 2.1% (2.0–2.2%) | 3.6% (3.5–3.7%) | 3.7% (3.6–3.8%) | 3.5% (3.4–3.6%) |
| Leafy Vegetables (Except Cabbages) | 0.3% (0.3–0.3%) | 0.3% (0.3–0.3%) | 0.4% (0.3–0.4%) | 0.6% (0.5–0.6%) | 0.5% (0.5–0.5%) | 0.6% (0.6–0.6%) |
| Fruiting Vegetables | 0.9% (0.8–0.9%) | 0.7% (0.7–0.7%) | 1.0% (0.9–1.0%) | 1.5% (1.4–1.5%) | 1.2% (1.2–1.3%) | 1.6% (1.6–1.7%) |
| Root Vegetables | 0.3% (0.3–0.3%) | 0.2% (0.2–0.2%) | 0.3% (0.3–0.4%) | 0.5% (0.5–0.5%) | 0.4% (0.4–0.4%) | 0.6% (0.5–0.6%) |
| Cabbages | 0.3% (0.3–0.3%) | 0.2% (0.2–0.2%) | 0.3% (0.3–0.3%) | 0.4% (0.4–0.5%) | 0.4% (0.4–0.4%) | 0.5% (0.5–0.5%) |
| Mushrooms | 0.1% (0.1–0.1%) | 0.1% (0.1–0.1%) | 0.1% (0.1–0.1%) | 0.1% (0.1–0.1%) | 0.1% (0.1–0.1%) | 0.1% (0.1–0.1%) |
| Grain And Pod Vegetables | 0.1% (0.1–0.1%) | 0.1% (0.0–0.1%) | 0.1% (0.1–0.1%) | 0.1% (0.1–0.1%) | 0.1% (0.1–0.1%) | 0.1% (0.1–0.1%) |
| Onion, Garlic | 0.1% (0.1–0.1%) | 0.1% (0.1–0.1%) | 0.1% (0.1–0.1%) | 0.2% (0.2–0.2%) | 0.2% (0.2–0.2%) | 0.2% (0.2–0.2%) |
| Stalk Vegetables, Sprouts | 0.2% (0.2–0.2%) | 0.2% (0.1–0.2%) | 0.2% (0.2–0.2%) | 0.3% (0.3–0.3%) | 0.3% (0.2–0.3%) | 0.4% (0.3–0.4%) |
| Mixed Salad, Mixed Vegetables | 0.3% (0.2–0.3%) | 0.2% (0.2–0.2%) | 0.3% (0.3–0.3%) | 0.4% (0.4–0.5%) | 0.4% (0.4–0.4%) | 0.5% (0.5–0.5%) |
| Legumes | 0.3% (0.3–0.3%) | 0.3% (0.3–0.4%) | 0.2% (0.2–0.3%) | 0.5% (0.5–0.5%) | 0.6% (0.6–0.6%) | 0.4% (0.4–0.4%) |
| Fruits | 2.0% (1.9–2.1%) | 1.5% (1.5–1.6%) | 2.4% (2.3–2.5%) | 3.4% (3.3–3.5%) | 2.7% (2.6–2.8%) | 3.9% (3.8–4.1%) |
| Nuts, Seeds, Nut Spread | 0.2% (0.2–0.2%) | 0.2% (0.2–0.2%) | 0.2% (0.2–0.2%) | 0.3% (0.3–0.4%) | 0.3% (0.3–0.4%) | 0.3% (0.3–0.4%) |
| Mixed Fruits | 0.1% (0.0–0.1%) | 0.1% (0.0–0.1%) | 0.1% (0.0–0.1%) | 0.1% (0.1–0.1%) | 0.1% (0.1–0.1%) | 0.1% (0.1–0.1%) |
| Milk | 2.4% (2.3–2.6%) | 2.1% (1.9–2.3%) | 2.7% (2.5–2.9%) | 4.0% (3.8–4.2%) | 3.5% (3.2–3.9%) | 4.4% (4.1–4.7%) |
| Milk Beverages | 0.6% (0.5–0.7%) | 0.6% (0.5–0.8%) | 0.6% (0.5–0.7%) | 0.9% (0.8–1.1%) | 1.0% (0.8–1.2%) | 0.9% (0.7–1.0%) |
| Yogurt | 3.5% (3.3–3.6%) | 2.6% (2.3–2.8%) | 4.1% (3.9–4.4%) | 5.7% (5.5–6.0%) | 4.4% (4.1–4.8%) | 6.8% (6.4–7.1%) |
| Fromage Blanc, Petits Suisses | 0.6% (0.6–0.6%) | 0.5% (0.4–0.5%) | 0.7% (0.7–0.8%) | 1.0% (1.0–1.1%) | 0.8% (0.8–0.9%) | 1.2% (1.1–1.3%) |
| Cheeses (Including Fresh Cheeses) | 1.2% (1.2–1.3%) | 1.1% (1.0–1.1%) | 1.4% (1.3–1.4%) | 2.1% (2.0–2.1%) | 1.8% (1.8–1.9%) | 2.2% (2.2–2.3%) |
| Cream Desserts, Puddings (Milk Based) | 0.5% (0.4–0.5%) | 0.5% (0.4–0.5%) | 0.5% (0.4–0.5%) | 0.8% (0.7–0.8%) | 0.8% (0.7–0.9%) | 0.8% (0.7–0.9%) |
| Dairy Creams | 0.1% (0.1–0.1%) | 0.1% (0.1–0.1%) | 0.1% (0.1–0.1%) | 0.1% (0.1–0.1%) | 0.1% (0.1–0.1%) | 0.1% (0.1–0.1%) |
| Flour, Flakes, Starches, Semolina | 0.1% (0.1–0.1%) | 0.1% (0.1–0.1%) | 0.1% (0.1–0.1%) | 0.2% (0.2–0.2%) | 0.2% (0.2–0.2%) | 0.2% (0.2–0.2%) |
| Rice | 3.9% (3.7–4.0%) | 3.5% (3.3–3.7%) | 4.2% (4.0–4.4%) | 6.6% (6.4–6.8%) | 6.3% (6.0–6.7%) | 6.8% (6.5–7.1%) |
| Pasta, Other Grain | 1.2% (1.2–1.3%) | 1.2% (1.2–1.3%) | 1.2% (1.2–1.3%) | 2.1% (2.0–2.1%) | 2.1% (2.0–2.2%) | 2.0% (2.0–2.1%) |
| Bread | 4.0% (3.9–4.1%) | 4.0% (3.9–4.2%) | 4.0% (3.9–4.1%) | 6.8% (6.6–6.9%) | 6.9% (6.7–7.2%) | 6.6% (6.4–6.8%) |
| Crispbread, Rusks | 0.2% (0.1–0.2%) | 0.2% (0.2–0.2%) | 0.1% (0.1–0.1%) | 0.3% (0.2–0.3%) | 0.3% (0.3–0.3%) | 0.2% (0.2–0.3%) |
| Breakfast Cereal | 0.4% (0.4–0.4%) | 0.4% (0.3–0.4%) | 0.4% (0.4–0.5%) | 0.7% (0.6–0.7%) | 0.6% (0.6–0.7%) | 0.7% (0.6–0.7%) |
| Salty Biscuits, Aperitif Biscuits | 0.1% (0.1–0.1%) | 0.1% (0.1–0.1%) | 0.1% (0.1–0.1%) | 0.1% (0.1–0.2%) | 0.2% (0.1–0.2%) | 0.1% (0.1–0.1%) |
| Dough And Pastry | 0.1% (0.1–0.1%) | 0.1% (0.1–0.1%) | 0.1% (0.1–0.1%) | 0.2% (0.2–0.2%) | 0.2% (0.2–0.2%) | 0.2% (0.2–0.2%) |
| Fresh Meat Unclassified | 0.0% (0.0–0.0%) | 0.0% (0.0–0.0%) | 0.0% (0.0–0.0%) | 0.0% (0.0–0.0%) | 0.0% (0.0–0.0%) | 0.0% (0.0–0.0%) |
| Beef | 0.3% (0.3–0.3%) | 0.4% (0.4–0.4%) | 0.3% (0.2–0.3%) | 0.5% (0.5–0.6%) | 0.7% (0.7–0.7%) | 0.4% (0.4–0.5%) |
| Veal | 0.0% (0.0–0.0%) | 0.0% (0.0–0.0%) | 0.0% (0.0–0.0%) | 0.0% (0.0–0.0%) | 0.1% (0.0–0.1%) | 0.0% (0.0–0.0%) |
| Pork | 0.4% (0.4–0.4%) | 0.5% (0.5–0.5%) | 0.3% (0.3–0.3%) | 0.7% (0.6–0.7%) | 0.8% (0.8–0.9%) | 0.5% (0.5–0.6%) |
| Mutton/Lamb | 0.0% (0.0–0.0%) | 0.0% (0.0–0.1%) | 0.0% (0.0–0.0%) | 0.1% (0.1–0.1%) | 0.1% (0.1–0.1%) | 0.1% (0.0–0.1%) |
| Unclassified And Other Poultry | 0.2% (0.2–0.2%) | 0.2% (0.2–0.2%) | 0.2% (0.2–0.2%) | 0.3% (0.3–0.4%) | 0.3% (0.3–0.4%) | 0.3% (0.3–0.4%) |
| Chicken, Hen | 0.2% (0.2–0.2%) | 0.2% (0.2–0.2%) | 0.2% (0.2–0.2%) | 0.3% (0.3–0.3%) | 0.4% (0.3–0.4%) | 0.3% (0.3–0.3%) |
| Rabbit (Domestic) | 0.0% (0.0–0.0%) | 0.0% (0.0–0.0%) | 0.0% (0.0–0.0%) | 0.0% (0.0–0.0% | 0.0% (0.0–0.0%) | 0.0% (0.0–0.0%) |
| Processed Meat | 1.4% (1.4–1.5%) | 1.6% (1.5–1.6%) | 1.3% (1.3–1.4%) | 2.5% (2.4–2.5%) | 2.8% (2.7–2.9%) | 2.2% (2.1–2.3%) |
| Offals | 0.0% (0.0–0.0%) | 0.0% (0.0–0.0%) | 0.0% (0.0–0.0%) | 0.1% (0.1–0.1%) | 0.1% (0.1–0.1%) | 0.1% (0.0–0.1%) |
| Fish | 33.8% (33.2–34.4%) | 34.7% (33.7–35.6%) | 33.1% (32.3–34.0%) | 2.2% (2.1–2.3%) | 2.3% (2.2–2.4%) | 2.1% (2.0–2.2%) |
| Fish Products, Fish in Crumbs | 9.1% (8.9–9.3%) | 9.8% (9.5–10.1%) | 8.5% (8.2–8.7%) | 0.6% (0.6–0.6%) | 0.7% (0.6–0.7%) | 0.6% (0.5–0.6%) |
| Egg | 0.1% (0.1–0.1%) | 0.1% (0.1–0.1%) | 0.1% (0.1–0.1%) | 0.2% (0.2–0.2%) | 0.2% (0.2–0.2%) | 0.2% (0.2–0.2%) |
| Unclassified | 0.0% (0.0–0.0%) | 0.0% (0.0–0.0%) | 0.0% (0.0–0.0%) | 0.0% (0.0–0.0%) | 0.1% (0.0–0.1%) | 0.0% (0.0–0.0%) |
| Vegetable Oils | 0.3% (0.3–0.3%) | 0.3% (0.3–0.3%) | 0.3% (0.3–0.4%) | 0.5% (0.5–0.6%) | 0.5% (0.5–0.5%) | 0.6% (0.5–0.6%) |
| Butter | 0.3% (0.3–0.3%) | 0.2% (0.2–0.3%) | 0.3% (0.3–0.3%) | 0.5% (0.5–0.5%) | 0.4% (0.4–0.5%) | 0.5% (0.5–0.6%) |
| Margarines | 0.1% (0.1–0.1%) | 0.1% (0.1–0.1%) | 0.1% (0.1–0.1%) | 0.2% (0.2–0.2%) | 0.2% (0.2–0.2%) | 0.2% (0.2–0.2%) |
| Sugar, Honey, Jam | 0.6% (0.6–0.7%) | 0.6% (0.6–0.7%) | 0.7% (0.6–0.7%) | 1.1% (1.0–1.1%) | 1.1% (1.0–1.2%) | 1.1% (1.0–1.2%) |
| Chocolate, Candy Bars, Paste | 0.7% (0.7–0.8%) | 0.7% (0.6–0.7%) | 0.7% (0.7–0.8%) | 1.2% (1.1–1.3%) | 1.2% (1.1–1.3%) | 1.2% (1.1–1.3%) |
| Confectionery Non-Chocolate | 0.0% (0.0–0.0%) | 0.0% (0.0–0.0%) | 0.0% (0.0–0.0%) | 0.1% (0.1–0.1%) | 0.0% (0.0–0.1%) | 0.1% (0.1–0.1%) |
| Ice Cream, Water Ice | 0.1% (0.1–0.1%) | 0.1% (0.1–0.1%) | 0.1% (0.1–0.1%) | 0.2% (0.2–0.2%) | 0.2% (0.2–0.2%) | 0.2% (0.2–0.2%) |
| Ice Cream | 0.0% (0.0–0.0%) | 0.0% (0.0–0.0%) | 0.0% (0.0–0.0%) | 0.0% (0.0–0.0%) | 0.0% (0.0–0.0%) | 0.0% (0.0–0.0%) |
| Cakes, Pies, Pastries | 1.3% (1.2–1.3%) | 1.2% (1.1–1.3%) | 1.3% (1.2–1.4%) | 2.1% (2.1–2.2%) | 2.1% (2.0–2.2%) | 2.2% (2.1–2.3%) |
| Unclassified | 0.2% (0.2–0.3%) | 0.3% (0.2–0.4%) | 0.1% (0.1–0.2%) | 0.4% (0.3–0.5%) | 0.6% (0.4–0.7%) | 0.2% (0.2–0.3%) |
| Fruit And Vegetable Juices | 2.9% (2.8–3.1%) | 2.7% (2.5–2.9%) | 3.1% (2.9–3.3%) | 4.9% (4.6–5.1%) | 4.7% (4.4–5.0%) | 5.0% (4.7–5.3%) |
| Carbonated, Soft, Isotonic | 0.5% (0.4–0.5%) | 0.5% (0.4–0.6%) | 0.4% (0.3–0.5%) | 0.8% (0.7–0.9%) | 0.9% (0.7–1.1%) | 0.7% (0.5–0.8%) |
| Coffee | 9.9% (9.5–10.2%) | 8.8% (8.4–9.3%) | 10.7% (10.2–11.1%) | 16.3% (15.8–16.7%) | 14.9% (14.3–15.5%) | 17.4% (16.7–18.0%) |
| Tea | 0.9% (0.9–1.0%) | 0.8% (0.7–0.9%) | 1.0% (0.9–1.1%) | 1.6% (1.5–1.7%) | 1.5% (1.3–1.6%) | 1.7% (1.5–1.9%) |
| Herbal Tea | 1.0% (0.9–1.1%) | 0.6% (0.5–0.7%) | 1.3% (1.2–1.4%) | 1.7% (1.5–1.8%) | 1.0% (0.9–1.2%) | 2.2% (2.0–2.3%) |
| Chicory, Substitutes | 0.0% (0.0–0.1%) | 0.0% (0.0–0.0%) | 0.1% (0.0–0.1%) | 0.1% (0.1–0.1%) | 0.0% (0.0–0.1%) | 0.1% (0.1–0.1%) |
| Waters | 1.5% (1.4–1.5%) | 1.2% (1.1–1.3%) | 1.7% (1.6–1.8%) | 3.5% (3.4–3.6%) | 2.9% (2.8–3.1%) | 4.0% (3.8–4.1%) |
| Wine | 4.5% (4.2–4.7%) | 5.0% (4.6–5.4%) | 4.1% (3.8–4.4%) | 7.6% (7.3–8.0%) | 8.7% (8.1–9.3%) | 6.8% (6.3–7.2%) |
| Beer, Cider | 2.8% (2.6–3.0%) | 5.0% (4.6–5.4%) | 1.1% (1.0–1.2%) | 4.8% (4.5–5.1%) | 8.6% (8.0–9.2%) | 1.8% (1.6–2.1%) |
| Unclassified And Other Sauces | 0.1% (0.1–0.1%) | 0.1% (0.1–0.1%) | 0.1% (0.1–0.1%) | 0.2% (0.2–0.2%) | 0.2% (0.2–0.2%) | 0.2% (0.2–0.2%) |
| Tomato Sauces | 0.0% (0.0–0.0%) | 0.0% (0.0–0.0%) | 0.0% (0.0–0.0%) | 0.1% (0.0–0.1%) | 0.0% (0.0–0.0%) | 0.1% (0.1–0.1%) |
| Dressing Sauces | 0.0% (0.0–0.0%) | 0.0% (0.0–0.0%) | 0.0% (0.0–0.0%) | 0.0% (0.0–0.0%) | 0.0% (0.0–0.0%) | 0.0% (0.0–0.0%) |
| Spices, Herbs and Flavorings | 0.0% (0.0–0.0%) | 0.0% (0.0–0.0%) | 0.0% (0.0–0.0%) | 0.0% (0.0–0.0%) | 0.0% (0.0–0.0%) | 0.0% (0.0–0.0%) |
| Condiments | 0.0% (0.0–0.0%) | 0.0% (0.0–0.0%) | 0.0% (0.0–0.0%) | 0.0% (0.0–0.0%) | 0.0% (0.0–0.0%) | 0.0% (0.0–0.0%) |
| Soups | 0.4% (0.4–0.5%) | 0.5% (0.5–0.6%) | 0.4% (0.3–0.4%) | 0.8% (0.7–0.8%) | 0.9% (0.9–1.0%) | 0.6% (0.6–0.7%) |
| Bouillon | 0.1% (0.0–0.1%) | 0.1% (0.1–0.1%) | 0.0% (0.0–0.1%) | 0.1% (0.1–0.1%) | 0.1% (0.1–0.1%) | 0.1% (0.1–0.1%) |
| Unclassified | 0.1% (0.1–0.1%) | 0.1% (0.1–0.1%) | 0.1% (0.1–0.1%) | 0.1% (0.1–0.2%) | 0.1% (0.1–0.1%) | 0.2% (0.1–0.2%) |
| Soya Products | 0.0% (0.0–0.0%) | 0.0% (0.0–0.0%) | 0.0% (0.0–0.0%) | 0.0% (0.0–0.0%) | 0.0% (0.0–0.0%) | 0.0% (0.0–0.0%) |

**Supplementary Table S3.** Sex-stratified distribution of dietary arsenic intake levels (µg/kg bw/d) by food type

| Food Group | tAs intake -Males | | tAs intake -Females | | Estimated iAs intake -Males | | Estimated iAs intake -Females | |
| --- | --- | --- | --- | --- | --- | --- | --- | --- |
|  | Min-Max | Median (IQR) | Min-Max | Median (IQR) | Min-Max | Median (IQR) | Min-Max | Median (IQR) |
| Potatoes | 6e-04–0.0596 | 0.01 (0.01) | 0–0.0984 | 0.01 (0.01) | 4e-04–0.0416 | 0.01 (0) | 0–0.0689 | 0.01 (0) |
| Leafy Vegetables (Except Cabbages) | 0–0.0187 | 0 (0) | 0–0.0463 | 0 (0) | 0–0.0131 | 0 (0) | 0–0.0324 | 0 (0) |
| Fruiting Vegetables | 0.0 - 0.0283 | 0 (0) | 3e-04–0.0652 | 0 (0) | 0–0.0199 | 0 (0) | 2e-04–0.0455 | 0 (0) |
| Root Vegetables | 0.0 - 0.0161 | 0 (0) | 0–0.0349 | 0 (0) | 0–0.0112 | 0 (0) | 0–0.0243 | 0 (0) |
| Cabbages | 0.0 - 0.0137 | 0 (0) | 0–0.0259 | 0 (0) | 0–0.0096 | 0 (0) | 0–0.0182 | 0 (0) |
| Mushrooms | 0.0 - 0.0081 | 0 (0) | 0–0.0047 | 0 (0) | 0–0.0057 | 0 (0) | 0–0.0033 | 0 (0) |
| Grain And Pod Vegetables | 0.0 - 0.0079 | 0 (0) | 0–0.0042 | 0 (0) | 0–0.0055 | 0 (0) | 0–0.003 | 0 (0) |
| Onion, Garlic | 0.0 - 0.0049 | 0 (0) | 0–0.005 | 0 (0) | 0–0.0035 | 0 (0) | 0–0.0035 | 0 (0) |
| Stalk Vegetables, Sprouts | 0.0 - 0–0.0134 | 0 (0) | 0–0.0277 | 0 (0) | 0–0.0094 | 0 (0) | 0–0.0194 | 0 (0) |
| Mixed Salad, Mixed Vegetables | 0–0.0113 | 0 (0) | 0–0.0106 | 0 (0) | 0–0.0079 | 0 (0) | 0–0.0074 | 0 (0) |
| Legumes | 0–0.0191 | 0 (0) | 0–0.0275 | 0 (0) | 0–0.0134 | 0 (0) | 0–0.0192 | 0 (0) |
| Fruits | 0–0.0688 | 0.01 (0.01) | 0–0.0762 | 0.01 (0.01) | 0–0.0482 | 0 (0) | 0–0.0533 | 0.01 (0.01) |
| Nuts, Seeds, Nut Spread | 0–0.0227 | 0 (0) | 0–0.04 | 0 (0) | 0–0.0159 | 0 (0) | 0–0.028 | 0 (0) |
| Mixed Fruits | 0–0.0054 | 0 (0) | 0–0.0093 | 0 (0) | 0–0.0037 | 0 (0) | 0–0.0064 | 0 (0) |
| Milk | 0–0.2298 | 0 (0.01) | 0–0.2194 | 0.01 (0.02) | 0–0.1609 | 0 (0.01) | 0–0.1536 | 0.01 (0.01) |
| Milk Beverages | 0–0.2089 | 0 (0) | 0–0.2309 | 0 (0) | 0–0.1463 | 0 (0) | 0–0.1616 | 0 (0) |
| Yogurt | 0–0.3811 | 0.01 (0.02) | 0–0.4007 | 0.01 (0.03) | 0–0.2668 | 0 (0.01) | 0–0.2805 | 0.01 (0.02) |
| Fromage Blanc, Petits Suisses | 0–0.0379 | 0 (0) | 0–0.0607 | 0 (0) | 0–0.0265 | 0 (0) | 0–0.0425 | 0 (0) |
| Cheeses (Including Fresh Cheeses) | 0–0.047 | 0 (0) | 0–0.0584 | 0.01 (0.01) | 0–0.0328 | 0 (0) | 0–0.0409 | 0 (0) |
| Cream Desserts, Puddings (Milk Based) | 0–0.0307 | 0 (0) | 0–0.0287 | 0 (0) | 0–0.0214 | 0 (0) | 0–0.0201 | 0 (0) |
| Dairy Creams | 0–0.0062 | 0 (0) | 0–0.0134 | 0 (0) | 0–0.0044 | 0 (0) | 0–0.0094 | 0 (0) |
| Flour, Flakes, Starches, Semolina | 0–0.0096 | 0 (0) | 0–0.0093 | 0 (0) | 0–0.0067 | 0 (0) | 0–0.0065 | 0 (0) |
| Rice | 0–0.3464 | 0.02 (0.02) | 0–0.4117 | 0.02 (0.02) | 0–0.2425 | 0.01 (0.01) | 0–0.2882 | 0.01 (0.01) |
| Pasta, Other Grain | 0–0.0576 | 0.01 (0) | 0–0.0479 | 0.01 (0.01) | 0–0.0403 | 0 (0) | 0–0.0336 | 0 (0) |
| Bread | 0–0.0965 | 0.02 (0.01) | 0–0.187 | 0.02 (0.01) | 0–0.0675 | 0.01 (0.01) | 0–0.1308 | 0.01 (0.01) |
| Crispbread, Rusks | 0–0.0191 | 0 (0) | 0–0.0308 | 0 (0) | 0–0.0134 | 0 (0) | 0–0.0215 | 0 (0) |
| Breakfast Cereals | 0–0.0308 | 0 (0) | 0–0.0549 | 0 (0) | 0–0.0216 | 0 (0) | 0–0.0384 | 0 (0) |
| Salty Biscuits, Aperitif Biscuits | 0–0.0235 | 0 (0) | 0–0.0159 | 0 (0) | 0–0.0165 | 0 (0) | 0–0.0111 | 0 (0) |
| Dough And Pastry | 0–0.0051 | 0 (0) | 0–0.01 | 0 (0) | 0–0.0035 | 0 (0) | 0–0.007 | 0 (0) |
| Fresh Meat Unclassified | 0–8e-04 | 0 (0) | 0–7e-04 | 0 (0) | 0–6e-04 | 0 (0) | 0–5e-04 | 0 (0) |
| Beef | 0–0.0201 | 0 (0) | 0–0.0205 | 0 (0) | 0–0.0141 | 0 (0) | 0–0.0144 | 0 (0) |
| Veal | 0–0.005 | 0 (0) | 0–0.0031 | 0 (0) | 0–0.0035 | 0 (0) | 0–0.0022 | 0 (0) |
| Pork | 0–0.0336 | 0 (0) | 0–0.018 | 0 (0) | 0–0.0236 | 0 (0) | 0–0.0125 | 0 (0) |
| Mutton/Lamb | 0–0.0061 | 0 (0) | 0–0.0041 | 0 (0) | 0–0.0042 | 0 (0) | 0–0.0029 | 0 (0) |
| Unclassified And Other Poultry | 0–0.0147 | 0 (0) | 0–0.0476 | 0 (0) | 0–0.0103 | 0 (0) | 0–0.0333 | 0 (0) |
| Chicken, Hen | 0–0.0152 | 0 (0) | 0–0.0147 | 0 (0) | 0–0.0106 | 0 (0) | 0–0.0103 | 0 (0) |
| Rabbit (Domestic) | 0–4e-04 | 0 (0) | 0–3e-04 | 0 (0) | 0–3e-04 | 0 (0) | 0–2e-04 | 0 (0) |
| Processed Meat | 0–0.0562 | 0.01 (0.01) | 0–0.0398 | 0.01 (0.01) | 0–0.0394 | 0.01 (0) | 0–0.0278 | 0 (0) |
| Offals | 0–0.0051 | 0 (0) | 0–0.0064 | 0 (0) | 0–0.0035 | 0 (0) | 0–0.0045 | 0 (0) |
| Fish | 0–1.7664 | 0.23 (0.28) | 0–8.8054 | 0.22 (0.26) | 0–0.0353 | 0 (0.01) | 0–0.1761 | 0 (0.01) |
| Fish Products, Fish in Crumbs | 0–0.9794 | 0.06 (0.07) | 0–6.2775 | 0.05 (0.06) | 0–0.0196 | 0 (0) | 0–0.1256 | 0 (0) |
| Egg | 0–0.0101 | 0 (0) | 0–0.0114 | 0 (0) | 0–0.0071 | 0 (0) | 0–0.0079 | 0 (0) |
| Unclassified | 0–0.0015 | 0 (0) | 0–0.0013 | 0 (0) | 0–0.001 | 0 (0) | 0–8e-04 | 0 (0) |
| Vegetable Oils | 0–0.0151 | 0 (0) | 0–0.0161 | 0 (0) | 0–0.0106 | 0 (0) | 0–0.0113 | 0 (0) |
| Butter | 0–0.018 | 0 (0) | 0–0.0129 | 0 (0) | 0–0.0125 | 0 (0) | 0–0.0091 | 0 (0) |
| Margarines | 0–0.0123 | 0 (0) | 0–0.0139 | 0 (0) | 0–0.0085 | 0 (0) | 0–0.0097 | 0 (0) |
| Sugar, Honey, Jam | 0–0.0728 | 0 (0) | 0–0.0722 | 0 (0) | 0–0.051 | 0 (0) | 0–0.0506 | 0 (0) |
| Chocolate, Candy Bars, Paste | 0–0.0849 | 0 (0) | 0–0.1024 | 0 (0) | 0–0.0595 | 0 (0) | 0–0.0717 | 0 (0) |
| Confectionery Non-Chocolate | 0–0.0048 | 0 (0) | 0–0.0086 | 0 (0) | 0–0.0033 | 0 (0) | 0–0.006 | 0 (0) |
| Ice Cream, Water Ice | 0–0.0087 | 0 (0) | 0–0.013 | 0 (0) | 0–0.0061 | 0 (0) | 0–0.0091 | 0 (0) |
| Ice Cream | 0–0.0017 | 0 (0) | 0–0.0023 | 0 (0) | 0–0.0012 | 0 (0) | 0–0.0016 | 0 (0) |
| Cakes, Pies, Pastries | 0–0.0775 | 0 (0.01) | 0–0.1131 | 0.01 (0.01) | 0–0.0543 | 0 (0) | 0–0.0792 | 0 (0) |
| Unclassified | 0–0.2699 | 0 (0) | 0–0.0949 | 0 (0) | 0–0.1889 | 0 (0) | 0–0.0665 | 0 (0) |
| Fruit And Vegetable Juices | 0–0.2214 | 0.01 (0.02) | 0–0.1853 | 0.01 (0.02) | 0–0.1549 | 0.01 (0.01) | 0–0.1297 | 0.01 (0.01) |
| Carbonated, Soft, Isotonic | 0–0.1481 | 0 (0) | 0–0.1506 | 0 (0) | 0–0.1037 | 0 (0) | 0–0.1054 | 0 (0) |
| Coffee | 0–0.4603 | 0.04 (0.05) | 0–0.6014 | 0.05 (0.06) | 0–0.3222 | 0.03 (0.04) | 0–0.421 | 0.03 (0.04) |
| Tea | 0–0.0705 | 0 (0.01) | 0–0.097 | 0 (0.01) | 0–0.0493 | 0 (0) | 0–0.0679 | 0 (0) |
| Herbal Tea | 0–0.0707 | 0 (0) | 0–0.1086 | 0 (0.01) | 0–0.0495 | 0 (0) | 0–0.076 | 0 (0.01) |
| Chicory, Substitutes | 0–0.0165 | 0 (0) | 0–0.034 | 0 (0) | 0–0.0116 | 0 (0) | 0–0.0238 | 0 (0) |
| Waters | 0–0.0382 | 0 (0.01) | 0–0.07 | 0.01 (0.01) | 0–0.0382 | 0 (0.01) | 0–0.07 | 0.01 (0.01) |
| Wine | 0–0.3896 | 0.01 (0.04) | 0–0.315 | 0.01 (0.03) | 0–0.2727 | 0.01 (0.03) | 0–0.2205 | 0.01 (0.02) |
| Beer, Cider | 0–0.4488 | 0.02 (0.03) | 0–0.2067 | 0 (0.01) | 0–0.3142 | 0.01 (0.02) | 0–0.1447 | 0 (0) |
| Unclassified And Other Sauces | 0–0.0073 | 0 (0) | 0–0.0067 | 0 (0) | 0–0.0051 | 0 (0) | 0–0.0047 | 0 (0) |
| Tomato Sauces | 0–0.004 | 0 (0) | 0–0.0074 | 0 (0) | 0–0.0029 | 0 (0) | 0–0.0052 | 0 (0) |
| Dressing Sauces | 0–0.0011 | 0 (0) | 0–6e-04 | 0 (0) | 0–8e-04 | 0 (0) | 0–4e-04 | 0 (0) |
| Spices, Herbs and Flavorings | 0–6e-04 | 0 (0) | 0–9e-04 | 0 (0) | 0–4e-04 | 0 (0) | 0–6e-04 | 0 (0) |
| Condiments | 0–4e-04 | 0 (0) | 0–7e-04 | 0 (0) | 0–3e-04 | 0 (0) | 0–5e-04 | 0 (0) |
| Soups | 0–0.0341 | 0 (0) | 0–0.0756 | 0 (0) | 0–0.0239 | 0 (0) | 0–0.0529 | 0 (0) |
| Bouillon | 0–0.0033 | 0 (0) | 0–0.0041 | 0 (0) | 0–0.0023 | 0 (0) | 0–0.0028 | 0 (0) |
| Unclassified | 0–0.0139 | 0 (0) | 0–0.0292 | 0 (0) | 0–0.0098 | 0 (0) | 0–0.0205 | 0 (0) |
| Soya Products | 0–0.001 | 0 (0) | 0–0.002 | 0 (0) | 0–7e-04 | 0 (0) | 0–0.0014 | 0 (0) |

**Supplementary Figure S7**. Estimated median inorganic arsenic intake across 17 food groups, stratified by sex

**Supplementary Table S4.** Correlation between total serum arsenic levels (µg/L) and dietary arsenic intake levels (µg/kg bw/d)

| Sex | Type of arsenic | Partial Spearman coefficient^a^ | 95% Cl | P value |
| --- | --- | --- | --- | --- |
| Overall | Total arsenic intake | 0.20 | 0.17-0.24 | <0.0001 |
| Overall | Estimated iAs intake | 0.07 | 0.03-0.11 | 0.00033 |
| Male | Total arsenic intake | 0.20 | 0.14–0.25 | <0.0001 |
| Female | Total arsenic intake | 0.21 | 0.16-0.26 | <0.0001 |
| Male | Estimated iAs intake | 0.07 | 0.01–0.13 | 0.02 |
| Female | Estimated iAs intake | 0.07 | 0.02–0.12 | 0.01 |

^a^ Adjusted for sex and age at recruitment

**Supplementary Table S5.** Correlation between total serum arsenic concentrations (µg/L) and dietary arsenic intake (µg/kg body weight/day) across 17 food groups and their subgroups

| Food GroupS | | Total Arsenic intake | | Estimated iAs intake | |
| --- | --- | --- | --- | --- | --- |
|  |  | ρ | p value | ρ^a^ | p value |
| 01 | Potatoes and tubers | 0.019 | 0.3226 | 0.019 | 0.3141 |
| 02 | Vegetables | 0.052 | 0.0074* | 0.051 | 0.0082* |
|  | Leafy vegetables (except cabbages) | 0.031 | 0.1129 | 0.033 | 0.0872 |
|  | Fruiting vegetables | 0.050 | 0.0094 | 0.049 | 0.0122 |
|  | Root vegetables | 0.002 | 0.9345 | 0.005 | 0.8161 |
|  | Cabbages | 0.019 | 0.3174 | 0.020 | 0.3013 |
|  | Mushrooms | 0.024 | 0.2497 | 0.028 | 0.1918 |
|  | Grain and pod vegetables | 0.012 | 0.5678 | 0.008 | 0.7351 |
|  | Onion, garlic | 0.067 | 0.0006 | 0.056 | 0.0049 |
|  | Stalk vegetables, sprouts | -0.002 | 0.9025 | -0.008 | 0.7062 |
|  | Mixed salad, mixed vegetables | 0.013 | 0.5046 | 0.007 | 0.7258 |
| 03 | Legumes | 0.001 | 0.9533 | -0.002 | 0.9171 |
| 04 | Fruits, nuts, seeds | 0.002 | 0.9149 | 0.003 | 0.8853 |
| 05 | Dairy products | -0.020 | 0.3025 | -0.020 | 0.3021 |
| 06 | Cereal and cereal products | -0.001 | 0.9422 | -0.001 | 0.9459 |
| 07 | Meat and meat products | 0.010 | 0.5893 | 0.012 | 0.5397 |
| 08 | Fish and shellfish | 0.211 | <0.0001* | 0.211 | <0.0001* |
|  | 0801 Fish | 0.209 | <0.0001* | 0.208 | <0.0001* |
|  | 0803 Fish products, fish in crumbs | 0.210 | <0.0001* | 0.210 | <0.0001* |
| 09 | Eggs and egg products | 0.032 | 0.1146 | 0.042 | 0.0535 |
| 10 | Fat | 0.027 | 0.1574 | 0.026 | 0.1784 |
| 11 | Sugar and confectionary | -0.032 | 0.1027 | -0.032 | 0.1050 |
| 12 | Cakes and biscuits | 0.004 | 0.8214 | 0.005 | 0.7948 |
| 13 | Non-alcoholic beverages | 0.013 | 0.5146 | 0.015 | 0.4318 |
| 14 | Alcoholic beverages | 0.125 | <0.0001* | 0.125 | <0.0001* |
|  | 1401 Wine | 0.110 | <0.0001* | 0.110 | <0.0001* |
|  | 1403 Beer, cider | 0.086 | 0.0001* | 0.086 | 0.0001* |
| 15 | Condiments and sauces | -0.040 | 0.0419* | -0.034 | 0.0828 |
| 16 | Soups, bouillons | 0.023 | 0.2364 | 0.025 | 0.2128 |
| 17 | Miscellaneous | 0.042 | 0.1738 | 0.033 | 0.2801 |

^a^ρ = Spearman`s rank correlation coefficient

**Supplementary Table S6**. Sensitivity analysis for the association between dietary arsenic intake concentrations (Median Upper Bound) and breast cancer risk by menopausal status at baseline (HR and 95%CI).

|  | Quartile of Arsenic | | | | *p*-Trend |
| --- | --- | --- | --- | --- | --- |
| Menopausal status at recruitment | Q1 | Q2 | Q3 | Q4 |  |
| Premenopausal: tAs intake |  |  |  |  |  |
| Number of cases | 38 | 56 | 54 | 63 |  |
| Sub-cohort non-cases | 147 | 146 | 148 | 145 |  |
| Median arsenic (µg/kg bw/d) | 0.34 | 0.50 | 0.68 | 0.96 |  |
| Crude HR (95% CI) | Ref | 1.37 (1.02–1.85) | 1.04 (0.77–1.4) | 1.13 (0.84–1.51) | 0.03 |
| Adjusted HR (95% CI)^a^ | Ref | 1.29 (0.93–1.78) | 1.06 (0.79–1.43) | 1.1 (0.81–1.48) | 0.09 |
| Postmenopausal: tAs intake |  |  |  |  |  |
| Number of cases | 82 | 78 | 74 | 75 |  |
| Sub-cohort non-cases | 208 | 218 | 215 | 216 |  |
| Median arsenic (µg/kg bw/d) | 0.32 | 0.49 | 0.67 | 0.96 |  |
| Crude HR (95% CI) | Ref | 0.92 (0.72–1.17) | 1.03 (0.8–1.31) | 1.02 (0.8–1.3) | 0.03 |
| Adjusted HR (95% CI)^a^ | Ref | 0.86 (0.67–1.12) | 1.02 (0.8–1.3) | 1.02 (0.79–1.3) | 0.10 |
| Premenopausal: Estimated iAs intake |  |  |  |  |  |
| Number of cases | 47 | 44 | 66 | 54 |  |
| Sub-cohort non-cases | 148 | 149 | 145 | 144 |  |
| Median arsenic (µg/kg bw/d) | 0.17 | 0.22 | 0.27 | 0.34 |  |
| Crude HR (95% CI) | Ref | 1.1 (0.81–1.48) | 0.92 (0.68–1.24) | 0.77 (0.58–1.03) | 0.62 |
| Adjusted HR (95% CI)^a^ | Ref | 0.96 (0.67–1.38) | 0.94 (0.7–1.27) | 0.8 (0.6–1.07) | 0.77 |
| Postmenopausal: Estimated iAs arsenic |  |  |  |  |  |
| Number of cases | 67 | 89 | 80 | 73 |  |
| Sub-cohort non-cases | 218 | 207 | 215 | 217 |  |
| Median arsenic (µg/kg bw/d) | 0.15 | 0.20 | 0.24 | 0.32 |  |
| Crude HR (95% CI) | Ref | 0.96 (0.74–1.24) | 0.88 (0.69–1.13) | 1.09 (0.86–1.38) | 0.62 |
| Adjusted HR (95% CI)^a^ | Ref | 0.87 (0.64–1.18) | 0.92 (0.72–1.18) | 1.05 (0.83–1.33) | 0.77 |

a Adjusted for level of education, BMI, smoking status, alcohol use, physical activity, vegetable and fruit intake, parity status, use of oral contraceptives/HRT, breastfeeding history, age at menarche, and family history of breast cancer

**Supplementary Table S7**. Sensitivity analysis for the association between dietary arsenic intake concentrations (Median Upper Bound) and breast cancer risk by menopausal status at diagnosis (HR and 95%CI).

|  | Quartile of Arsenic | | | | *p*-Trend |
| --- | --- | --- | --- | --- | --- |
| Menopausal status at diagnosis | Q1 | Q2 | Q3 | Q4 |  |
| Premenopausal: tAs intake |  |  |  |  |  |
| Number of cases | 15 | 18 | 22 | 34 |  |
| Sub-cohort non-cases | 422 | 432 | 430 | 424 |  |
| Median arsenic (µg/kg bw/d) | 0.33 | 0.50 | 0.68 | 0.97 |  |
| Crude HR (95% CI) | Ref | 1.24 (0.91–1.69) | 1.34 (0.97–1.86) | 0.95 (0.67–1.34) | 0.1 |
| Adjusted HR (95% CI)^a^ | Ref | 1.2 (0.86–1.67) | 1.37 (0.99–1.91) | 0.93 (0.66–1.31) | 0.16 |
| Postmenopausal: tAs intake |  |  |  |  |  |
| Number of cases | 130 | 148 | 130 | 134 |  |
| Sub-cohort non-cases | 420 | 431 | 429 | 425 |  |
| Median arsenic (µg/kg bw/d) | 0.33 | 0.50 | 0.67 | 0.96 |  |
| Crude HR (95% CI) | Ref | 1.02 (0.85–1.22) | 0.97 (0.81–1.17) | 1.1 (0.92–1.32) | 0.86 |
| Adjusted HR (95% CI)^a^ | Ref | 1 (0.82–1.21) | 0.97 (0.81–1.16) | 1.1 (0.92–1.32) | 0.97 |
| Premenopausal: Estimated iAs intake |  |  |  |  |  |
| Number of cases | 14 | 11 | 33 | 31 |  |
| Sub-cohort non-cases | 431 | 425 | 426 | 426 |  |
| Median arsenic (µg/kg bw/d) | 0.15 | 0.20 | 0.25 | 0.33 |  |
| Crude HR (95% CI) | Ref | 1.08 (0.78–1.51) | 1.03 (0.74–1.42) | 0.87 (0.63–1.21) | 0.79 |
| Adjusted HR (95% CI)^a^ | Ref | 1.01 (0.68–1.5) | 1.03 (0.74–1.43) | 0.87 (0.63–1.21) | 0.97 |
| Postmenopausal: Estimated iAs intake |  |  |  |  |  |
| Number of cases | 126 | 145 | 141 | 130 |  |
| Sub-cohort non-cases | 430 | 422 | 427 | 429 |  |
| Median arsenic (µg/kg bw/d) | 0.15 | 0.20 | 0.25 | 0.33 |  |
| Crude HR (95% CI) | Ref | 1.03 (0.86–1.24) | 0.95 (0.79–1.14) | 1.01 (0.84–1.21) | 0.79 |
| Adjusted HR (95% CI)^a^ | Ref | 1.01 (0.81–1.26) | 0.96 (0.8–1.15) | 0.98 (0.82–1.17) | 0.97 |

^a^ Covariates included in the models were consistent with those specified in Model 6, unless otherwise indicated. Hazard ratios (HRs) and 95% confidence intervals (CIs) were estimated using Cox proportional hazards regression models.

**Supplementary Table S8.** Sensitivity analysis of the association between dietary arsenic intake concentrations (median upper bound) and breast cancer risk by estrogen receptor/progesterone receptor (ER/PR) status (HR [95% CI]).

|  | Quartile of Arsenic | | | | *p*-Trend |
| --- | --- | --- | --- | --- | --- |
| Receptor status | Q1 | Q2 | Q3 | Q4 |  |
| ER/PR+: tAs intake |  |  |  |  |  |
| Number of cases | 107 | 117 | 105 | 120 |  |
| Sub-cohort non-cases | 422 | 432 | 430 | 424 |  |
| Median arsenic (µg/kg bw/d) | 0.33 | 0.50 | 0.67 | 0.97 |  |
| Crude HR (95% CI) | Ref | 1.07 (0.88–1.29) | 1.1 (0.91–1.34) | 1.11 (0.91–1.35) | 0.39 |
| Adjusted HR (95% CI)^a^ | Ref | 1.02 (0.84–1.26) | 1.09 (0.89–1.32) | 1.11 (0.91–1.35) | 0.67 |
| ER/PR-: tAs intake |  |  |  |  |  |
| Number of cases | 19 | 30 | 23 | 28 |  |
| Sub-cohort non-cases | 422 | 432 | 430 | 424 |  |
| Median arsenic (µg/kg bw/d) | 0.33 | 0.50 | 0.67 | 0.97 |  |
| Crude HR (95% CI) | Ref | 1.06 (0.78–1.45) | 1.19 (0.86–1.64) | 1.06 (0.76–1.48) | 0.58 |
| Adjusted HR (95% CI)^a^ | Ref | 1.09 (0.78–1.51) | 1.2 (0.87–1.66) | 1.04 (0.74–1.45) | 0.52 |
| ER/PR+: Estimated iAs intake |  |  |  |  |  |
| Number of cases | 101 | 119 | 120 | 109 |  |
| Sub-cohort non-cases | 431 | 425 | 426 | 426 |  |
| Median arsenic (µg/kg bw/d) | 0.15 | 0.20 | 0.25 | 0.33 |  |
| Crude HR (95% CI) | Ref | 1.03 (0.84–1.25) | 0.98 (0.81–1.19) | 0.99 (0.82–1.2) | 0.82 |
| Adjusted HR (95% CI)^a^ | Ref | 0.95 (0.75–1.2) | 0.98 (0.81–1.2) | 0.96 (0.79–1.17) | 0.66 |
| ER/PR-: Estimated iAs intake |  |  |  |  |  |
| Number of cases | 19 | 13 | 41 | 27 |  |
| Sub-cohort non-cases | 431 | 425 | 426 | 426 |  |
| Median arsenic (µg/kg bw/d) | 0.15 | 0.20 | 0.25 | 0.33 |  |
| Crude HR (95% CI) | Ref | 1.14 (0.82–1.6) | 0.91 (0.65–1.26) | 0.81 (0.59–1.11) | 0.24 |
| Adjusted HR (95% CI)^a^ | Ref | 1.27 (0.85–1.9) | 0.9 (0.65–1.25) | 0.8 (0.58–1.1) | 0.28 |

^a^ Covariates included in the models were consistent with those specified in Model 6, unless otherwise indicated. Hazard ratios (HRs) and 95% confidence intervals (CIs) were estimated using Cox proportional hazards regression models.

**Supplementary Table S9.** Sensitivity analysis of the association between dietary arsenic intake concentrations (median upper bound) and breast cancer risk by human epidermal growth factor receptor 2 (HER2) status (HR [95% CI]).

|  | Quartile of Arsenic | | | | *p*-Trend |
| --- | --- | --- | --- | --- | --- |
| Receptor status | Q1 | Q2 | Q3 | Q4 |  |
| HER2-Positive: tAs intake |  |  |  |  |  |
| Number of cases | 19 | 20 | 22 | 28 |  |
| Sub-cohort non-cases | 422 | 432 | 430 | 424 |  |
| Median arsenic (µg/kg bw/d) | 0.33 | 0.50 | 0.67 | 0.96 |  |
| Crude HR (95% CI) | Ref | 1.12 (0.83–1.53) | 1.29 (0.94–1.79) | 1.01 (0.72–1.41) | 0.33 |
| Adjusted HR (95% CI)^a^ | Ref | 1.15 (0.83–1.59) | 1.33 (0.96–1.85) | 0.99 (0.71–1.39) | 0.28 |
| HER2-Negative: tAs intake |  |  |  |  |  |
| Number of cases | 95 | 112 | 97 | 108 |  |
| Sub-cohort non-cases | 422 | 432 | 430 | 424 |  |
| Median arsenic (µg/kg bw/d) | 0.33 | 0.50 | 0.67 | 0.97 |  |
| Crude HR (95% CI) | Ref | 1.04 (0.85–1.27) | 1.08 (0.88–1.32) | 1.1 (0.9–1.35) | 0.57 |
| Adjusted HR (95% CI)^a^ | Ref | \|0.99 (0.8–1.23) | 1.06 (0.87–1.3) | 1.1 (0.89–1.35) | 0.93 |
| HER2-Positive: Estimated iAs intake |  |  |  |  |  |
| Number of cases | 20 | 15 | 27 | 27 |  |
| Sub-cohort non-cases | 431 | 425 | 426 | 426 |  |
| Median arsenic (µg/kg bw/d) | 0.15 | 0.20 | 0.25 | 0.33 |  |
| Crude HR (95% CI) | Ref | 1.19 (0.86–1.64) | 1.07 (0.77–1.47) | 0.91 (0.66–1.26) | 0.29 |
| Adjusted HR (95% CI)^a^ | Ref | 1.33 (0.9–1.94) | 1.08 (0.78–1.49) | 0.91 (0.66–1.26) | 0.14 |
| HER2-Negative: Estimated iAs intake |  |  |  |  |  |
| Number of cases | 86 | 104 | 122 | 100 |  |
| Sub-cohort non-cases | 431 | 425 | 426 | 426 |  |
| Median arsenic (µg/kg bw/d) | 0.15 | 0.20 | 0.25 | 0.33 |  |
| Crude HR (95% CI) | Ref | 1.07 (0.87–1.32) | 0.9 (0.73–1.1) | 0.96 (0.79–1.17) | 0.63 |
| Adjusted HR (95% CI)^a^ | Ref | 0.99 (0.77–1.27) | 0.9 (0.73–1.1) | 0.93 (0.76–1.14) | 0.80 |

^a^ Covariates included in the models were consistent with those specified in Model 6, unless otherwise indicated. Hazard ratios (HRs) and 95% confidence intervals (CIs) were estimated using Cox proportional hazards regression models.

**Supplementary Table S10.** Sensitivity analysis for the association between dietary arsenic intake concentrations (Median lower Bound) and breast cancer in the EPIC-Heidelberg case–cohort (HR and 95%CI).

|  | Quartile of Arsenic | | | | *p*-Trend |
| --- | --- | --- | --- | --- | --- |
| Dietary Arsenic intake | Q1 | Q2 | Q3 | Q4 |  |
| Total arsenic intake |  |  |  |  |  |
| Number of cases | 149 | 171 | 140 | 171 |  |
| Sub-cohort non-cases | 422 | 431 | 429 | 426 |  |
| Median arsenic (µg/kg bw/d) | 0.08 | 0.22 | 0.37 | 0.61 |  |
| Crude HR (95% CI) | Ref | 1.09 (0.92–1.3) | 1.03 (0.87–1.23) | 1.14 (0.96–1.36) | 0.25 |
| Adjusted HR (95% CI)^a^ | Ref | 1.07 (0.9–1.28) | 1.03 (0.87–1.22) | 1.14 (0.96–1.35) | 0.34 |
| Estimated iAs intake |  |  |  |  |  |
| Number of cases | 161 | 156 | 136 | 178 |  |
| Sub-cohort non-cases | 425 | 422 | 430 | 431 |  |
| Median arsenic (µg/kg bw/d) | 0.01 | 0.02 | 0.03 | 0.05 |  |
| Crude HR (95% CI) | Ref | 1.1 (0.93–1.3) | 1.18 (1–1.4) | 0.15 (0.96–1.37) | 0.12 |
| Adjusted HR (95% CI)^a^ | Ref | 1.05 (0.89–1.26) | 1.16 (0.98–1.38) | 0.14 (0.95–1.36) | 0.32 |

^a^ Covariates included in the models were consistent with those specified in Model 6, unless otherwise indicated. Hazard ratios (HRs) and 95% confidence intervals (CIs) were estimated using Cox proportional hazards regression models.

**Supplementary Table S11.** Sensitivity analysis for the association between dietary arsenic intake concentrations (Mean upper and lower Bound) and breast cancer in the EPIC-Heidelberg case–cohort (HR and 95% CI).

|  | Quartile of Arsenic | | | | *p*-Trend |
| --- | --- | --- | --- | --- | --- |
| Dietary Arsenic Mean Upper and Lower Bound | Q1 | Q2 | Q3 | Q4 |  |
| Mean Upper Bound: tAs intake |  |  |  |  |  |
| Number of cases | 146 | 162 | 157 | 166 |  |
| Sub-cohort non-cases | 424 | 429 | 431 | 424 |  |
| Median arsenic (µg/kg bw/d) | 0.45 | 0.67 | 0.89 | 1.26 |  |
| Crude HR (95% CI) | Ref | 1.08 (0.91–1.28) | 0.99 (0.83–1.17) | 1.07 (0.9–1.26) | 0.39 |
| Adjusted HR (95% CI)^a^ | Ref | 1.05 (0.88–1.25) | 1 (0.84–1.18) | 1.08 (0.91–1.28) | 0.59 |
| Mean Upper Bound: Estimated iAs intake |  |  |  |  |  |
| Number of cases | 139 | 158 | 175 | 159 |  |
| Sub-cohort non-cases | 431 | 424 | 427 | 426 |  |
| Median arsenic (µg/kg bw/d) | 0.22 | 0.29 | 0.36 | 0.47 |  |
| Crude HR (95% CI) | Ref | 1.03 (0.86–1.22) | 0.91 (0.76–1.08) | 0.94 (0.8–1.11) | 0.88 |
| Adjusted HR (95% CI)^a^ | Ref | 0.97 (0.79–1.18) | 0.92 (0.77–1.09) | 0.93 (0.79–1.11) | 0.62 |
| Mean Lower Bound: tAs intake |  |  |  |  |  |
| Number of cases | 160 | 159 | 140 | 172 |  |
| Sub-cohort non-cases | 421 | 432 | 430 | 425 |  |
| Median arsenic (µg/kg bw/d) | 0.18 | 0.37 | 0.56 | 0.85 |  |
| Crude HR (95% CI) | Ref | 1.07 (0.9–1.26) | 1.1 (0.93–1.3) | 1.08 (0.91–1.29) | 0.36 |
| Adjusted HR (95% CI)^a^ | Ref | 1.05 (0.88–1.24) | 1.11 (0.93–1.31) | 1.08 (0.91–1.28) | 0.45 |
| Mean Lower Bound: Estimated iAs intake |  |  |  |  |  |
| Number of cases | 156 | 139 | 180 | 156 |  |
| Sub-cohort non-cases | 428 | 422 | 423 | 435 |  |
| Median arsenic (µg/kg bw/d) | 0.06 | 0.09 | 0.11 | 0.16 |  |
| Crude HR (95% CI) | Ref | 1.01 (0.85–1.2) | 0.97 (0.82–1.15) | 0.89 (0.75–1.06) | 0.97 |
| Adjusted HR (95% CI)^a^ | Ref | 0.94 (0.78–1.14) | 0.98 (0.82–1.16) | 0.91 (0.77–1.08) | 0.47 |

^a^ Covariates included in the models were consistent with those specified in Model 6, unless otherwise indicated. Hazard ratios (HRs) and 95% confidence intervals (CIs) were estimated using Cox proportional hazards regression models.

**Supplementary Table S12.** Sensitivity analysis for the association between dietary arsenic intake concentrations and all-cause mortality in the EPIC-Heidelberg case–cohort (HR and 95%CI).

|  | Quartile of Arsenic | | | | p-Trend |
| --- | --- | --- | --- | --- | --- |
| Dietary Arsenic | Q1 | Q2 | Q3 | Q4 |  |
| Median Upper Bound: tAs intake |  |  |  |  |  |
| Number of deaths | 52 | 42 | 47 | 46 |  |
| Sub-cohort non-cases | 412 | 422 | 419 | 421 |  |
| Median arsenic (µg/kg bw/d) | 0.33 | 0.50 | 0.67 | 0.97 |  |
| Crude HR (95% CI) | Ref | 0.89 (0.62–1.28) | 0.94 (0.65–1.34) | 1.01 (0.71–1.45) | 0.51 |
| Adjusted HR (95% CI)^a^ | Ref | 0.91 (0.62–1.34) | 0.95 (0.65–1.37) | 0.95 (0.66–1.36) | 0.61 |
| Median Upper Bound: Estimated iAs intake |  |  |  |  |  |
| Number of deaths | 54 | 56 | 44 | 33 |  |
| Sub-cohort non-cases | 404 | 414 | 426 | 430 |  |
| Median arsenic (µg/kg bw/d) | 0.15 | 0.20 | 0.25 | 0.33 |  |
| Crude HR (95% CI) | Ref | 0.86 (0.57–1.28) | 0.67 (0.46–0.97) | 1.06 (0.75–1.49) | 0.12 |
| Adjusted HR (95% CI)^a^ | Ref | 0.93 (0.59–1.46) | 0.6 (0.41–0.87) | 1.12 (0.79–1.58) | 0.32 |
| Median Lower Bound: tAs intake |  |  |  |  |  |
| Number of cases | 52 | 45 | 43 | 47 |  |
| Sub-cohort non-cases | 411 | 422 | 420 | 421 |  |
| Median arsenic (µg/kg bw/d) | 0.45 | 0.67 | 0.89 | 1.25 |  |
| Crude HR (95% CI) | Ref | 0.86 (0.6–1.24) | 0.89 (0.62–1.28) | 1.09 (0.76–1.56) | 0.40 |
| Adjusted HR (95% CI)^a^ | Ref | 0.91 (0.62–1.34) | 0.9 (0.62–1.3) | 1.02 (0.71–1.46) | 0.59 |
| Median Lower Bound: Estimated iAs intake |  |  |  |  |  |
| Number of cases | 55 | 52 | 48 | 32 |  |
| Sub-cohort non-cases | 404 | 416 | 423 | 431 |  |
| Median arsenic (µg/kg bw/d) | 0.22 | 0.29 | 0.36 | 0.47 |  |
| Crude HR (95% CI) | Ref | 0.79 (0.53–1.17) | 0.73 (0.5–1.06) | 0.81 (0.57–1.15) | 0.26 |
| Adjusted HR (95% CI)^a^ | Ref | 0.86 (0.55–1.35) | 0.67 (0.46–0.97) | 0.85 (0.6–1.21) | 0.51 |

^a^ Covariates included in the models were consistent with those specified in Model 6, unless otherwise indicated. Hazard ratios (HRs) and 95% confidence intervals (CIs) were estimated using Cox proportional hazards regression models.
